# Rab4A organizes endosomal domains for sorting cargo to lysosome-related organelles

**DOI:** 10.1242/jcs.216226

**Published:** 2018-09-20

**Authors:** Sudeshna Nag, Shikha Rani, Sarmistha Mahanty, Christin Bissig, Pooja Arora, Cristina Azevedo, Adolfo Saiardi, Peter van der Sluijs, Cedric Delevoye, Guillaume van Niel, Graca Raposo, Subba Rao Setty

**Affiliations:** 1 Department of Microbiology and Cell Biology, Indian Institute of Science, Bangalore, India 560 012; 2 Institut Curie, PSL Research University, CNRS, UMR 144, Structure and Membrane Compartments, F-75005, Paris, France; 3 Medical Research Council Laboratory for Molecular Cell Biology, University College London, Gower Street, London, WC1E 6BT, UK; 4 Cellular Protein Chemistry, Bijvoet Center for Biomolecular Research, Utrecht University, 3584 CH Utrecht, The Netherlands; 5 Institut Curie, PSL Research University, CNRS, UMR144, Cell and Tissue Imaging Facility (PICT-IBiSA), F-75005, Paris, France

**Keywords:** Rab4A, AP-3, KIF3, Rabenosyn-5, Rabaptin-5, Rabip4′, Sorting endosome, Melanosome biogenesis

## Abstract

Sorting endosomes (SEs) are the regulatory hubs for sorting cargo to multiple organelles, including lysosome-related organelles, such as melanosomes in melanocytes. In parallel, melanosome biogenesis is initiated from SEs with the processing and sequential transport of melanocyte-specific proteins toward maturing melanosomes. However, the mechanism of cargo segregation on SEs is largely unknown. Here, RNAi screening in melanocytes revealed that knockdown of Rab4A results in defective melanosome maturation. Rab4A-depletion increases the number of vacuolar endosomes and disturbs the cargo sorting, which in turn lead to the mislocalization of melanosomal proteins to lysosomes, cell surface and exosomes. Rab4A localizes to the SEs and forms an endosomal complex with the adaptor AP-3, the effector rabenosyn-5 and the motor KIF3, which possibly coordinates cargo segregation on SEs. Consistent with this, inactivation of rabenosyn-5, KIF3A or KIF3B phenocopied the defects observed in Rab4A-knockdown melanocytes. Further, rabenosyn-5 was found to associate with rabaptin-5 or Rabip4/4′ (isoforms encoded by *Rufy1*) and differentially regulate cargo sorting from SEs. Thus, Rab4A acts a key regulator of cargo segregation on SEs.

This article has an associated First Person interview with the first author of the paper.

## Abstract (precis)

**Summary:** Rab4A controls cargo segregation by forming a complex with rabenosyn-5, KIF3 and AP-3 on sorting endosomes to divert the cargo toward melanosomes.

## INTRODUCTION

Organelles of the endocytic system constantly mature into terminal organelles, such as lysosomes in all cells and lysosome-related organelles (LROs) in specialized cells (Luzio et al., 2014; Raposo et al., 2007). Melanosomes are the LROs of melanocytes present in the skin and eye, which provide color and photoprotection. These organelles are derived by post-sequential trafficking of multiple melanosomal cargoes from sorting/recycling endosomes (SEs/REs) to maturing melanosomes (Marks et al., 2013; Sitaram and Marks, 2012). For example, pre-melanosomal protein (PMEL) is segregated into intraluminal vesicles (ILVs) of SEs (also called stage I melanosomes) for fibril formation (conversion from stage I to II) (van Niel et al., 2011); tyrosinase-related protein-1 (TYRP1), the copper transporter ATP7A and other cargo are sorted into RE tubular structures by a BLOC-1-dependent transport mechanism (Setty et al., 2007, 2008); and tyrosinase (TYR) is sorted into endosomal vesicles by the AP-3-dependent transport pathway (Theos et al., 2005) on SEs, which are then targeted to stage II melanosomes for melanin synthesis (conversion from stage II to stage III and IV) (Marks et al., 2013). This process is essential for the step-wise maturation of melanosomes from stage I to IV to avoid pigment formation in SEs. However, the mechanism of melanocytic cargo sorting on SEs is poorly understood.

Domain organization and cargo sorting on endocytic membranes are predicted to be mediated by Rab GTPases (Rabs) and adaptor proteins (APs) (Bonifacino and Lippincott-Schwartz, 2003; Stenmark, 2009; Zerial and McBride, 2001). In general, Rabs recruit effector proteins, including kinesin motors and SNAREs, during vesicle budding/transport and membrane fusion, respectively (Bonifacino and Glick, 2004; Ohbayashi and Fukuda, 2012; Pfeffer, 2013). In contrast, APs such as the AP-3 and AP-1 complexes have been shown to segregate both melanocytic and non-melanocytic cargoes on endosomes by binding to unique amino acid motifs in the cargo tails (Bonifacino and Traub, 2003; Park and Guo, 2014). Studies using live-cell imaging have reported the existence of AP-1- and AP-3-independent domains on SEs (D'Souza et al., 2014). Moreover, the tetraspanin-like protein CD63 in melanocytes or Cos proteins in yeast have been shown to regulate sorting of multiple cargoes on SEs (MacDonald et al., 2015; van Niel et al., 2011). Nevertheless, the mechanism by which melanosomal cargoes are segregated into subdomains on SEs is poorly studied. Additionally, several Rabs, such as Rab4, Rab5, Rab7, Rab9, Rab11 and Rab22 (Grant and Donaldson, 2009; Pfeffer, 2013; Stenmark, 2009; Zerial and McBride, 2001), and their multiple effectors or tethering proteins, such as rabenosyn-5, rabaptin-5, Rabip4′ (the longer isoform of Rabip4; encoded by *Rufy1*), EEA1 and the HOPS complex, have been shown to localize to these domains (de Renzis et al., 2002; Deneka et al., 2003; Fouraux et al., 2004; Ivan et al., 2012; Kalin et al., 2015; Zhu et al., 2009) for the regulation of different cargo transport processes. Furthermore, Rab7 and Rab9 have been shown to control different transport steps during melanosome biogenesis by functioning on late endosomes (LEs) or melanosomes (Hida et al., 2011; Mahanty et al., 2016). Nonetheless, the function of Rab4A in regulating the trafficking of melanocytic cargo during melanosome maturation has not been studied.

Rab4A has been shown to be involved in many cellular processes, including fast recycling of cargo to the cell surface (Mohrmann et al., 2002; van der Sluijs et al., 1992) and conversion of Rab5-postive early endosomes (EEs) into Rab11-positive REs (de Renzis et al., 2002; Sönnichsen et al., 2000). Interestingly, Rab4A independently binds to the Rab5A effectors such as rabenosyn-5, rabaptin-5 and Rabip4′, as well as to the AP-1 and AP-3 complexes (D'Souza et al., 2014; de Renzis et al., 2002; Deneka et al., 2003; Fouraux et al., 2004; Ivan et al., 2012; Vitale et al., 1998). However, the specific step of cargo trafficking/sorting in which Rab4A interacts with these multiple molecules is unclear. Moreover, Rab4A co-fractionates with the kinesin-2 motor protein KIF3 (heterodimer of KIF3A and KIF3B) and regulates endosomal positioning/distribution (Bananis et al., 2004). Recently, Rab4 has been shown to associate with either the KIF3A or KIF13A motors on anterograde transport vesicles in *Drosophila* and regulate synapse organization (Dey et al., 2017). Nevertheless, the importance of the Rab4A–KIF3 interaction in endosomal organization or its role in organelle biogenesis has not been well studied. Rab4A has also been shown to modulate autophagy directly (Talaber et al., 2014) or in response to mechanical membrane stretch (Yao et al., 2016), and has a role in exocytosis of phagosomes containing pathogenic bacteria (Takeuchi et al., 2015). Taken together, these studies suggest that Rab4A either participates in multiple pathways by interacting with different effectors or forms a unique protein complex assembled on the endosomal membrane that regulates different transport steps.

In this study, we aimed to dissect the role of Rab4A in melanosome biogenesis by taking advantage of the well-known melanocytic cargo transport steps between SEs/REs and maturing melanosomes. Our studies provide evidence that Rab4A acts as a key regulator in sorting multiple cargoes on SEs through forming a unique protein complex with AP-3, rabenosyn-5 and KIF3A/B. Moreover, this complex associates with rabaptin-5 to sort PMEL to stage II melanosomes and with Rabip4 and/or Rabip4′ (hereafter Rabip4/4′) to sort TYRP1 and TYR to REs in melanocytes. Importantly, our study show that the absence of Rab4A expression blocks melanosome maturation at stage II, upregulates melanophagosome formation and alters cargo sorting into exosomes. Thus, Rab4A is essentially required for cargo segregation on SEs, which occurs possibly through creating different endosomal domains using its multiple effector molecules.

## RESULTS

### Rab4A is required for cargo sorting on SEs and melanocyte pigmentation

SEs act as the key intermediary organelles during the biogenesis of melanosomes in melanocytes (Bissig et al., 2016; Jani et al., 2016; Marks et al., 2013) in addition to their role in cargo transport to the cell surface, Golgi or lysosomes, which is similar to their role in other mammalian cells (Grant and Donaldson, 2009; Klumperman and Raposo, 2014). On the SE membrane, multiple melanocyte-specific cargoes must be segregated and transported through different routes to the melanosomes during their sequential maturation from stage I to IV. However, the mechanism of cargo segregation on SEs is poorly understood. We hypothesized that Rab GTPases had a role in this process, and performed an RNAi screen using shRNAs (transfected transiently, denoted as sh) against endosomal/late endosomal Rab proteins (Rab3A, Rab4A, Rab4B, Rab5A, Rab5B, Rab5C, Rab7A and Rab11A) in wild-type (WT) mouse melanocytes (melan-Ink) (Fig. S1A,B). We confirmed the gene knockdown (we observed 30–40% of transcript depletion except in the case of Rab5B sh, Fig. S1C) and analyzed the cells for following cellular phenotype. We predicted that the reduced Rab expression would cause mislocalization of melanocytic cargoes to the lysosomes for degradation following hypopigmentation of melanocytes. Visual quantification of pigmentation loss by bright-field microscopy (BFM) showed that more than 40% of Rab3A-, 4A-, 5A-, 7A- and 11A-depleted melanocytes had a hypopigmentation phenotype when compared to control cells (Fig. S1A, gray bars). Quantitative immunofluorescence microscopy (IFM) showed reduced TYRP1 and TYR intensities (indicative of their lysosomal degradation) in Rab3A, 4A-, 5A- and 11A-depleted melanocytes (Fig. S1A). Among these, Rab4A and Rab11A (but not Rab5A)-knockdown melanocytes displayed reduced levels of melanin content compared to control cells, and their respective protein levels were also reduced in these cells (Fig. S1D). In addition, another melanosomal protein, PMEL, was mislocalized to lysosomes in Rab4A-depleted melanocytes compared to control or other Rab-inactivated cells (Fig. S1B). Thus, we wanted to evaluate the role of Rab4A in the cargo transport pathways to melanosome.

**Fig. 1.**
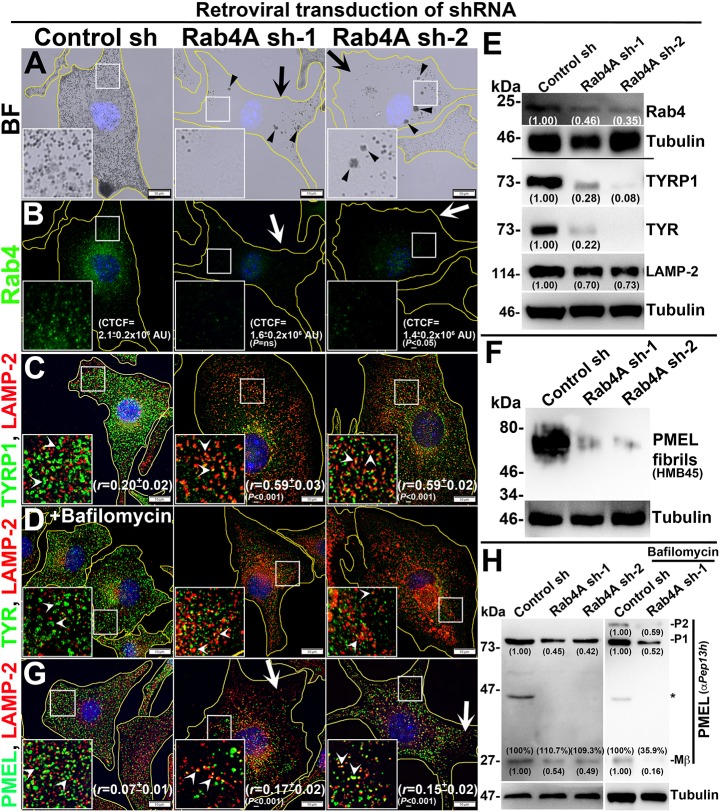
**Rab4A-knockdown affects melanocyte pigmentation and cargo transport to melanosomes.** BF (A) and IFM (B–D,G) images of Rab4A-depleted (sh-1 and sh-2) and control melanocytes. Black arrows and arrowheads indicate the pigmentation loss and melanosome clusters, respectively (A). White arrows indicate the loss in fluorescence staining of Rab4 (B) or PMEL (G) in knockdown cells. White arrowheads (C,D,G) point to the cargo localization to lysosomes. In D, cells were treated with bafilomycin. Nuclei are stained with Hoechst 33258. The insets are a magnified view of the white boxed areas. The Pearson's coefficient (*r*) between the two markers and CTCF values are indicated separately (mean±s.e.m.). Scale bars: 10 µm. (E,F,H) Immunoblotting analysis of Rab4, melanosomal and lysosomal proteins, and PMEL fibrils in Rab4A-knockdown cells. Tubulin was used as a loading control. P1, P2 and Mβ, are the full-length, glycosylated ER form and processed PMEL forms. *non-specific band. Relative protein band intensities were quantified and are indicated on the gels.

Retroviral transduction of WT melanocytes with two different shRNAs (sh-1 and sh-2) specific to mouse Rab4A caused a severe pigmentation defect compared to control shRNA-transduced melanocytes (Fig. 1A, arrows). Additionally, a large number of melanosome clusters (MCs) that resembled the melanophagosomes (Boissy et al., 1987) (Fig. 1A, arrowheads) were also observed in Rab4A-depleted melanocytes (see below). Estimation of the amount of melanin pigment in Rab4A-knockdown cells showed a moderate reduction in melanin content compared to control melanocytes (Fig. S1E). However, a visual quantification of the number of pigmented melanocytes during four independent experiments (similar to Fig. 1A) revealed that ∼80% of cells were hypopigmented in Rab4A-inactivated conditions compared to ∼20% in control conditions (Fig. S1F). IFM and biochemical analyses showed that Rab4 staining (Fig. 1B), transcript (Fig. S1G) and protein levels (see Fig. 1E) were dramatically reduced in Rab4A-knockdown compared to control cells. Consistent with this, the corrected total cell fluorescence (CTCF) of Rab4 staining in Rab4A-depleted cells was notably reduced compared to control melanocytes (CTCF=1.6±0.2×10^6^ AU, not significant in Rab4A sh-1; 1.4±0.2×10^6^ AU, *P*≤0.05 in Rab4A sh-2; compared to 2.1±0.2×10^6^ AU in control cells) (Fig. 1B). We also found that although the gross localization of Rab5 was unaffected, its fluorescence intensity was slightly reduced in the peripheral cytosol of Rab4A-depleted melanocytes (arrows, Fig. S1H). Furthermore, rescuing of Rab4A-depleted cells with GFP–Rab4A (susceptible to Rab4A shRNA) marginally restored the melanocyte pigmentation and cargo (TYRP1) stability (Fig. S1Ia). In line with this, transfection of Rab4A sh-2-knockdown melanocytes with GFP–Rab4A^sh2R^ (resistant to shRNA and localizes similarly to Rab4A^WT^; data not shown) rescued melanocyte pigmentation and restored the TYRP1 protein levels and its localization to melanosomes (Fig. S1Ib, data not shown for cargo levels). These results indicate that the phenotypes observed in Rab4A sh cells are specific to the Rab4A depletion. IFM analysis further revealed that the fluorescence intensities of both melanosome-localized TYRP1 and TYR were dramatically reduced in Rab4A-depleted melanocytes compared to control melanocytes (Fig. 1C; Fig. S1J). In addition, localization of the remaining TYRP1 appeared as punctate structures that colocalized significantly with LAMP-2-positive compartments in the Rab4A-knockdown melanocytes (*r*=0.59±0.03, *P*≤0.001 in Rab4A sh-1; 0.59±0.02, *P*≤0.001 in Rab4A sh-2; compared to 0.20±0.02 in control cells) (Fig. 1C). Moreover, the localization of TYRP1 to LAMP-2-positive structures was further enhanced upon treatment of bafilomycin A1 (a vacuolar ATPase inhibitor) in Rab4A sh cells compared to control sh cells (see Fig. S1L), indicating that TYRP1 is targeted for lysosomal degradation upon Rab4A depletion in melanocytes. In contrast, TYR appeared as diffused a cytosolic signal and its localization to LAMP-2-positive compartments was slightly restored upon treatment with bafilomycin in Rab4A-depleted cells (Fig. S1J; Fig. 1D). Consistent with these results, the activity of TYR, as measured through a 3,4-dihydroxyphenylalanine (DOPA) assay, was completely abolished in Rab4A shRNA-transduced cells compared to control cells (Fig. S1K). As expected, both TYRP1 and TYR were localized as ring-like structures (Fig. 1C; Fig. S1J) that were positive for melanosomes by BFM (data not shown) in control cells. Immunoblotting analysis showed that both TYRP1 and TYR protein levels were dramatically reduced and nearly restored to that of control cells upon lysosomal inhibition with bafilomycin in Rab4A-depleted melanocytes (Fig. 1E; Fig. S1M). These studies indicate that Rab4A inactivation in melanocytes results in hypopigmentation due to mistargeting of both TYRP1 and TYR to lysosomes.

Next, we examined whether Rab4A-knockdown affects formation of PMEL fibrils and maturation of stage II melanosomes in melanocytes. IFM staining of PMEL (using the HMB45 antibody) in Rab4A-depleted melanocytes was dramatically reduced (arrows) and remaining PMEL partially colocalized with LAMP-2-positive lysosomes (arrowheads) compared to control cells (*r*=0.17±0.02, *P*≤0.001 in Rab4A sh-1; 0.15±0.02, *P*≤0.001 in Rab4A sh-2; compared to 0.07±0.01 in control cells) (Fig. 1G). Consistent with these results, the total PMEL fibrils isolated from Rab4A-knockdown cells was drastically reduced compared to control cells (Fig. 1F). Previous studies have shown that PMEL (the P1 form) undergoes endosomal processing into Mα, Mβ and C-terminal fragment (CTF) forms (which can be detected with anti-Pep13h antibody as shown in Rochin et al., 2013), and Mα further matures into fibrils in SEs/stage I melanosomes (Bissig et al., 2016). Immunoblotting of Rab4A-knockdown cells showed reduced levels of both the P1 and Mβ forms compared to control cells (Fig. 1H). Surprisingly, the percentage of Mβ generated from the total PMEL was not affected in Rab4A-depleted compared to control cells (Fig. 1H), suggesting that proteolytic processing of PMEL is not affected upon Rab4A inactivation. Furthermore, the decreased levels of P1 and Mβ in Rab4A-depleted cells were not restored in the presence of bafilomycin (Fig. 1H) or protease inhibitors (data not shown), indicating that PMEL is not targeted for lysosomal degradation; however, a portion of PMEL was mislocalized onto lysosomal membranes (Fig. 1G,H). These results encouraged us to investigate the mislocalization of PMEL to other organelles. It is most likely that unprocessed PMEL would be segregated into the ILVs of LEs and then secreted as exosomes upon Rab4A depletion in melanocytes. As predicted, the amount of the P1 form of PMEL was notably increased in the exosomes derived from Rab4A-knockdown melanocytes compared to control cells (Fig. S1N). Moreover, a small portion of Rab4 was associated with the exosomes released from the control cells (Fig. S1N), similar to a previous study (Vidal and Stahl, 1993). These studies suggest that Rab4A depletion in melanocytes causes mislocalization of full-length PMEL to the lysosomes and exosomes, which possibly reduces the total amount of cellular fibrils.

The sorting of proteolytically processed PMEL into ILVs is partially dependent on the non-melanocytic cargo CD63 (van Niel et al., 2011). We therefore examined whether Rab4A knockdown affects trafficking of other endocytic cargoes, such as CD63, LAMP-1, LAMP-2 and the transferrin receptor (TfR), from SEs in melanocytes. IFM analysis showed that localization of GFP–CD63 to lysosomes was moderately, although not significantly, increased in Rab4A-depleted melanocytes compared to control cells (*r*=0.40±0.04 in Rab4A sh-1 compared to 0.30±0.05 in control cells) (Fig. S1O). As expected, the colocalization between PMEL and GFP–CD63 was also increased in these cells (*r*=0.49±0.03, *P*≤0.001 in Rab4A sh-1 compared to 0.23±0.04 in control cells) (Fig. S1O). Further, quantitative IFM colocalization experiments showed that a pool of lysosomal proteins, such as LAMP-2, was accumulated in the endosomal compartments upon Rab4A-knockdown compared to control melanocytes (Fig. S1P). Consistent with this, the protein levels of LAMP-2 were slightly reduced in Rab4A-knockdown cells (Fig. 1E). In contrast, the cell surface expression of LAMP-1, but not TfR, was moderately increased, similar to what was found for the melanocytic cargo PMEL and TYRP1 (despite a slight increase in the total protein levels of LAMP-1 and TfR) in Rab4A shRNA cells compared to control cells (Fig. S1Q). These studies indicate a slight defect in the trafficking of lysosomal proteins but not fast recycling cargoes upon Rab4A depletion in melanocytes. Overall, these studies illustrate that Rab4A controls trafficking of structural melanosome cargoes from SEs to melanosomes, and thus is a critical regulator of melanosome biogenesis.

### Rab4A depletion in melanocytes causes an accumulation of enlarged endosomes and alters the stages of melanosome biogenesis

**Fig. 2.**
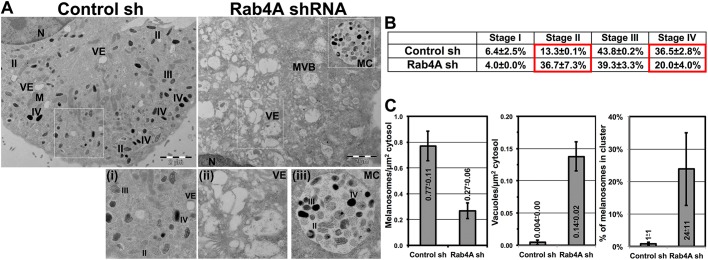
**Rab4A depletion increases the number of vacuolar endosomes and inhibits melanosome maturation in melanocytes.** (A–C) Electron microscopy analysis of control and Rab4A-knockdown melanocytes. M, mitochondria; MVB, multi-vesicular bodies; MC, melanosome cluster; N, nucleus; VE, vacuolar endosomes; II/III/IV, stages of melanosomes. Scale bars: 2 µm. (B) Percentage of each melanosome stage (mean±s.e.m., quantified as Materials and Methods) for both conditions. Significant changes in the values are highlighted in red box. (C) Melanosomes or vacuoles per µm^2^ of cytosol, or percentage of melanosomes in a cluster (mean±s.e.m.) for both the conditions.

Rab4A depletion possibly alters endosomal morphology/dynamics in WT melanocytes and thus causes mislocalization of the melanocytic cargo. Electron microscopy (EM) analysis showed an increase in the number of enlarged vacuolar structures in Rab4A-knockdown melanocytes (Fig. 2A, right panel and inset ii), whereas, in control melanocytes, all melanosome biogenesis stages (II to IV) were observed (Fig. 2A, left panel and inset i). Quantification of the data demonstrated that percentage of stage II melanosomes was dramatically increased (mostly present in the phagosomes, see below) in Rab4A-depleted compared to control melanocytes (13.3±0.1% in control shRNA and 36.7±7.3% in Rab4A shRNA cells) (Fig. 2B). Concurrently, the percentage of stage IV melanosomes was notably reduced (including the melanosomes present in phagosomes, see below) in Rab4A-knockdown compared to control cells (Fig. 2B). As expected, the number of melanosomes or vacuoles per µm^2^ of cytosol was reciprocal in Rab4A shRNA compared to control shRNA cells (Fig. 2C). Surprisingly, melanosomes in Rab4A-knockdown cells formed clusters (referred to here as MCs) resembling melanophagosome structures (Fig. 2A, inset iii) (Boissy et al., 1987), similar to the clusters observed in cells using BFM (arrowheads, Fig. 1A). Moreover, IFM analysis showed a portion of the LC3-positive puncta (but not LAMP-2) were associated with these MCs (Fig. S1R). Notably, the percentage of melanosomes in MCs was increased in Rab4A-depleted cells compared to that in control melanocytes (Fig. 2C). Consistent with these results, the cultured medium turned black during the initial stages (2 to 3 days) of Rab4A knockdown, which did not occur with control melanocytes (data not shown), suggesting that these MCs possibly undergo exocytosis. However, the mechanism by which Rab4A depletion increases the formation of these MCs is unknown. We hypothesized that Rab4A-deficiency possibly alters the autophagy in these cells (Talaber et al., 2014; Yao et al., 2016). These results indicate that Rab4A knockdown results in enlarged endosomes that likely alter the cargo segregation on SEs and the numbers of melanosome intermediates.

### Rab4A associates with Rab5A-shared effectors and provides the specificity to cargo segregation on SE membranes

**Fig. 3.**
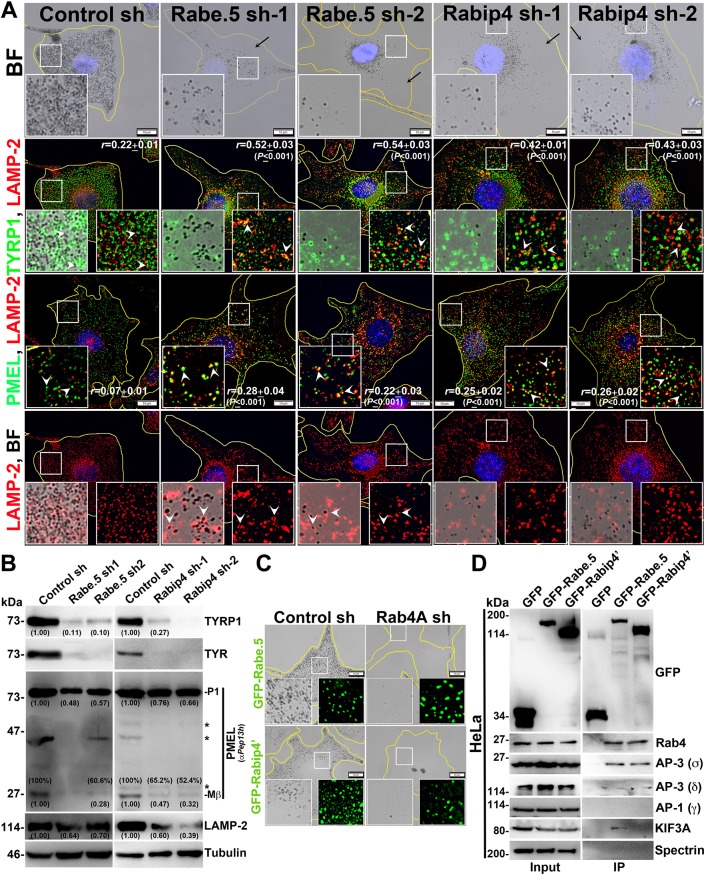
**Rabenosyn-5 and Rabip4 regulate the cargo trafficking to melanosomes, and rabenosyn-5 forms a complex with Rab4A–AP-3–KIF3.** (A,C) BF and IFM analysis of rabenosyn-5 (Rabe.5)- or Rabip4-knockdown cells (sh-1 and sh-2), or GFP–rabenosyn-5 and GFP–Rabip4′ expression in Rab4A-depleted melanocytes. Black arrows indicate the loss in pigmentation and arrowheads point to the cargo localization to lysosomes or melanosomes. The colocalization coefficient (*r*) between the proteins is indicated separately. Nuclei are stained with Hoechst 33258. The insets are a magnified view of the white boxed areas. Scale bars: 10 µm. (B) Immunoblotting analysis of melanosomal and lysosomal proteins in knockdown cells. Tubulin was used as a loading control. P1 and Mβ, full-length and processed PMEL bands. *non-specific bands. Protein band intensities were quantified and are indicated on the gels. (D) IP of GFP–rabenosyn-5, GFP–Rabip4′ and GFP (control) in HeLa cells. Both cell lysate (input) and IP blots were probed as indicated. Spectrin was used as a positive control for IP.

As observed, Rab4A regulates trafficking of different melanosomal and lysosomal cargoes; however, Rab4A alone may not be sufficient to segregate the cargo into subdomains on SEs. We hypothesized that effectors that act with both between Rab4A and Rab5A (referred to here as Rab4A-Rab5A-shared effectors) possibly play a role during this process. Studies have shown that Rab4A associates with many endosomal effectors in which rabenosyn-5, Rabip4/4′ and rabaptin-5 are either recruited by or associate with Rab5A on endosomal membranes (Fig. S2A) (de Renzis et al., 2002; Fouraux et al., 2004; Mattera et al., 2003). Additionally, rabenosyn-5 and Rabip4/4′ possess a FYVE domain (conserved in Fab1, YOTB, Vac1 and EEA1 proteins) that binds to phosphatidylinositol 3-phosphate (PI3P) lipids on the endosomal membranes (Mari et al., 2001; Nielsen et al., 2000). Nevertheless, the role of these shared effectors in cargo transport to melanosomes is unknown. Sequential knockdown of individual effectors resulted in a severe hypopigmentation defect similar to what was seen in Rab4A-depleted melanocytes (Fig. 3A; Fig. S2B). Furthermore, IFM analysis of rabenosyn-5-knockdown or Rabip4-knockdown (depleting both Rabip4 and Rabip4′ isoforms) melanocytes showed a dramatic loss in peripheral staining of TYRP1 and a pool was targeted to lysosomes for degradation (Fig. 3A) [*r*=0.52±0.03 in rabenosyn-5 sh-1; 0.54±0.03 in rabenosyn-5 sh-2; 0.42±0.01 in Rabip4 sh-1; 0.43±0.03 in Rabip4 sh-2 (all *P*≤0.001); compared to 0.22±0.01 in control cells]. In contrast, TYRP1 in rabaptin-5-depleted melanocytes was moderately affected and a small population was targeted to lysosomes (Fig. S2B) [*r*=0.47±0.02 in rabaptin-5 sh-1; 0.44±0.03 in rabaptin-5 sh-2 (both *P*≤0.001); compared to 0.22±0.01 in control cells]. In line with these data, immunoblot analysis showed that TYRP1 levels were dramatically reduced in rabenosyn-5- or Rabip4-knockdown cells but moderately decreased in rabaptin-5-knockdown cells (Fig. 3B; Fig. S2C) compared to respective control melanocytes. Similarly, the protein levels of TYR (an AP-3-dependent cargo) were also reduced, similar to what was found for TYRP1 levels, in the respective effector-knockdown cells (Fig. 3B; Fig. S2C). This data suggests that both rabenosyn-5 and Rabip4/4′ either separately or cooperatively regulate the initial segregation of these cargoes on SEs.

Interestingly, PMEL fluorescence intensity was dramatically reduced and a pool was targeted to lysosomes in all effector-depleted melanocytes (Fig. 3A; Fig. S2B), similar to what was seen in Rab4A-knockdown melanocytes (Fig. 1G). Consistent with this result, the P1 form of PMEL was drastically reduced in rabenosyn-5 shRNA cells (Fig. 3B), similar to what was seen in Rab4A shRNA cells (Fig. 1H), and was moderately decreased in Rabip4 shRNA or rabaptin-5 shRNA cells (Fig. 3B; Fig. S2C). In contrast to Rab4A-depleted melanocytes, the percentage of Mβ generated after proteolytic processing was drastically affected in rabaptin-5-knockdown cells and moderately affected in both rabenosyn-5- and Rabip4-depleted melanocytes (Fig. 3B; Fig. S2C), suggesting that rabaptin-5 likely regulates either PMEL segregation on SEs or its maturation in melanocytes. In line with these results, the amount of fibrils isolated from the rabenosyn-5-depleted or rabaptin-5-knockdown (data not shown) melanocytes was notably reduced compared to control cells, similar to in Rab4A-depleted melanocytes (Fig. 1F). Similarly, the fluorescence intensity and protein level of lysosomal membrane protein LAMP-2 were also dramatically reduced in both rabenosyn-5- and Rabip4- depleted, but not rabaptin-5-depleted melanocytes (Fig. 3A,B, Fig. S2B,C). Moreover, LAMP-2 was partially mislocalized to melanosomes in rabenosyn-5- and rabaptin-5-knockdown cells, indicating that rabenosyn-5 and rabaptin-5 either individually or jointly regulate the trafficking of lysosomal proteins and PMEL. Overall, these studies illustrate that rabenosyn-5-depleted cells share the phenotypes of both Rabip4- and rabaptin-5-knockdown melanocytes. Based on these data, we hypothesized (see below) that Rabip4/4′, in association with rabenosyn-5, possibly regulates the segregation/sorting of TYRP1 and TYR cargo, whereas rabaptin-5, in association with rabenosyn-5, likely controls the processing or maturation of PMEL fibrils in melanocytes.

### Rab4A-Rab5A-shared effectors interact with each other and are independently recruited to the endosomal membranes upstream of Rab4A

We further examined whether any of the Rab4A-Rab5A-shared effectors can rescue the hypopigmentation defect of Rab4A-depleted melanocytes. Unexpectedly, expression of none of these effectors (GFP–rabenosyn-5, GFP–Rabip4′ or mCherry–rabaptin-5) individually improved the pigmentation defect of Rab4A-inactivated melanocytes (Fig. 3C; Fig. S2D). This result suggests that Rab4A co-ordinates these effectors by functioning downstream in the trafficking pathway (Kalin et al., 2015). Furthermore, we analyzed the stability and localization of these effector proteins in different knockdown melanocytes to understand their molecular regulation. Upon Rab4A depletion, the Rabip4 level, but not that of rabenosyn-5 or rabaptin-5, were moderately reduced in melanocytes (Fig. S3A). Similarly, rabenosyn-5-depleted melanocytes displayed reduced levels of Rab4 and Rabip4, but not rabapatin-5, compared to control cells (Fig. S3A). In contrast, knockdown of either Rabip4 or rabaptin-5 did not change the protein levels of other effectors, including Rab4 levels (Fig. S3A). These studies indicate that rabenosyn-5 mediates the molecular interaction between these effectors. Similar to what is seen in fibroblasts, the GFP- or mCherry-tagged shared effectors localized as punctate structures (de Renzis et al., 2002; Fouraux et al., 2004; Mattera et al., 2003), with the pattern resembling EEA1-positive early endosomes in WT melanocytes (Setty et al., 2007), and colocalized with Rab5-positive endosomes (Fig. S3B, inset for GFP–rabenosyn-5 and data not shown for others). IFM studies showed that GFP–rabenosyn-5 (but not GFP–Rabip4′ or mCherry–rabaptin-5)-positive punctate structures were dispersed throughout the cell upon Rab4A depletion in melanocytes (Fig. S3B). In contrast, a reduced number of GFP–Rabip4′ punctate structures (in the periphery, arrows) and an increased cytosolic signal of mCherry–rabaptin-5 (arrows) was observed in rabenosyn-5-depleted melanocytes. In line with these results, the distribution of GFP–rabenosyn-5-positive punctate structures was moderately affected in both Rabip4- and rabaptin-5-depleted melanocytes (Fig. S3B). Taken together, these studies indicate that rabenosyn-5 regulates the localization of Rabip4/4′ and rabaptin-5 in melanocytes. Consistent with this conclusion, subcellular fractionation revealed that distribution of Rabip4/4′ to multiple membrane fractions and the localization of rabaptin-5 to the cytosol was increased in rabenosyn-5-knockdown melanocytes (Fig. S3C). Thus, rabenosyn-5 plays a key role in regulating the localization of Rabip4/4′ and rabaptin-5 to specific membranes. Based on this regulation, we predicted that rabenosyn-5 could interact with Rabip4/4′ or rabaptin-5 either in a complex or independently. Owing to low plasmid transfection efficiency of melanocytes, we immunoprecipitated Rabip4′ from HeLa cells expressing GFP–Rabip4′, which showed an interaction with rabenosyn-5 (Fig. S3D), but not with rabaptin-5 (data not shown). This may be due to the low endogenous expression of rabaptin-5 in HeLa cells (Fig. S3E). However, the expression of rabaptin-5 in melanocytes was considerably higher than in HeLa cells (Fig. S3E). Immunoprecipitation of endogenous rabaptin-5 revealed no interaction with either rabenosyn-5 or Rabip4, but strong binding to Rab4 in melanocyte lysates (Fig. S3F) (Kalin et al., 2015; Mattera et al., 2003), indicating that interaction between rabaptin-5 and other effectors is likely to be very transient in nature. Overall, these results suggest that the recruitment of Rab4A-Rab5A-shared effectors to endosomal membranes is independent of Rab4A and possibly function upstream of Rab4A. Moreover, rabenosyn-5 regulates recruitment of rabaptin-5, the stability/membrane distribution of Rabip4/4′ and cargo transport to melanosomes.

### Rab4A associates with rabenosyn-5 and coordinates cargo sorting by forming a rabenosyn5–KIF3–AP-3 complex

To understand the molecular regulation between Rab4A and the shared effectors in controlling cargo sorting on SEs, we studied the large-scale interactome of rabenosyn-5 and Rabip4′ in HeLa cells owing to their high plasmid transfection efficiency compared to melanocytes (data not shown). Interestingly, the shared effectors showed an interaction with endosomal adaptor proteins (AP-3/AP-1) and the kinesin-2 family motor KIF3. To validate these interactions, we performed immunoprecipitation (IP) of GFP–rabenosyn-5, GFP–Rabip4′ or GFP (as a control) using HeLa cell lysates. GFP–rabenosyn-5 showed a strong interaction with the Rab4, AP-3 and KIF3A, but not with AP-1 (Fig. 3D). However, GFP–Rabip4′ interacted only with the Rab4 and AP-3 (Ivan et al., 2012), but not with KIF3A or AP-1 (Fig. 3D). We predicted that the Rabip4′–Rab4–AP-3 interaction is possibly mediated through rabenosyn-5, since Rabip4′ and rabenosyn-5 associate with each other (Fig. S3D). Moreover, we hypothesize that a similar molecular interactions exists in melanocytes. To validate whether rabenosyn-5–Rab4A–AP-3–KIF3A complex localize to the endosomal membranes in melanocytes, we carried out subcellular fractionation. Upon the membrane fractionation of WT melanocytes Rab4, KIF3A, rabenosyn-5 and AP-3 (δ and σ) molecules segregated into the same membrane fractions, namely 5 to 7 (Fig. S4A, quantified and plotted as a graph in Fig. S4B). As observed, these fractions were positive for several organelle-specific markers, such as Rab5 (EEs), STX13 (also known as STX12) (REs) and LIMPII (late endosomes and Golgi), but not for LAMP-2 (lysosomes), suggesting that the Rab4A–rabenosyn-5-associated complex localizes to the endosomal membranes of the EE/SE/RE/LE (Fig. S4A). Interestingly, the membrane association of this complex was independent of AP-3, since the Rab4A–rabenosyn-5-associated complex fractionated to the same membranes (fractions 5 to 7) even in the absence of AP-3 expression in melanocytes (Fig. S4A, AP-3^−^, melan-mh cells-deficient for δ subunit) (Jani et al., 2015), suggesting that these molecules associate to the membranes independently of AP-3 recruitment. This is possibly due to the interaction of the rabenosyn-5 FYVE domain with PI3P on endosomal membranes (Nielsen et al., 2000). Thus, these results identified a new endosomal complex, Rab4A–rabenosyn-5–KIF3A–AP-3, that possibly controls the segregation and trafficking of multiple cargoes on SEs. Moreover, co-IP of endogenous Rab4A showed an interaction with AP-3 (and also with AP-1), KIF3A and KIF3B but not with Rab4A-Rab5A-shared effectors in melanocytes (Fig. S4C), indicating that the association of these dual effectors with the complex is very transient in nature. However, the role of KIF3A, but not AP-3 (Theos et al., 2005), in cargo sorting at SEs or in melanosome biogenesis is unknown.

**Fig. 4.**
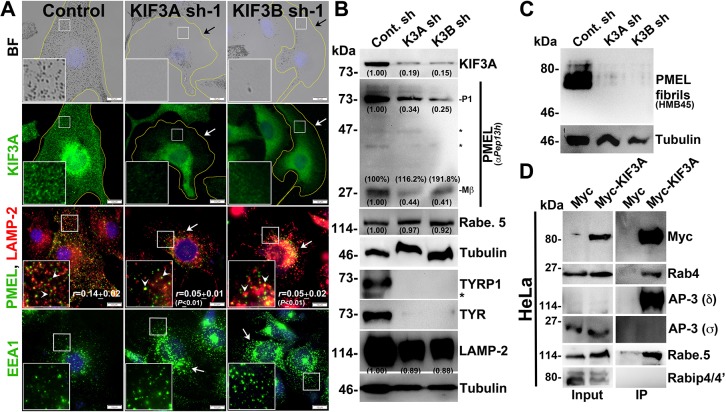
**KIF3 regulates melanocyte pigmentation and forms a complex with Rab4A–rabenosyn-5–AP-3.** (A) BF and IFM analysis of KIF3A or KIF3B-knockdown melanocytes. Black arrows indicate the loss of pigmentation. White arrows show the loss in KIF3A or PMEL staining, or clustering of EEA1 in KIF3A or KIF3B sh cells. White arrowheads point to the colocalization of PMEL with LAMP-2. Their colocalization coefficient (*r*) is indicated separately. Nuclei are stained with Hoechst 33258. The insets are a magnified view of the white boxed areas. Scale bars: 10 µm. (B,C) Immunoblotting analysis of melanosomal and lysosomal proteins, and PMEL fibrils in KIF3A or KIF3B-knockdown cells. Tubulin was used as a loading control. P1 and Mβ, full length and processed PMEL bands. *non-specific bands. Protein band intensities were quantified and are indicated on the gels. (D) Immunoprecipitation of Myc–KIF3A in HeLa cells. Both input and IP blots were probed as indicated.

Previous studies have shown that KIF3A (of the kinesin-2 family) and KIF5B (of the kinesin-1 family) localize to the Rab4A-positive early endosomal membrane fractions (Bananis et al., 2004). Studies have also shown that KIF3A interacts with AP-3β1 to mediate the release of HIV-1 Gag protein (Azevedo et al., 2009) and form a heterotrimeric complex with KIF3B (Yamazaki et al., 1995) and KAP3 (Yamazaki et al., 1996). We tested whether KIF3A or KIF3B has any role in cargo trafficking to melanosomes. Depletion of either KIF3A or KIF3B in WT melanocytes resulted in severe loss in pigmentation (Fig. 4A). As expected, KIF3A fluorescence staining and protein levels were reduced in KIF3A or KIF3B-depleted melanocytes (Fig. 4A,B). Similar to what is seen with Rab4A-depleted melanocytes, KIF3A- and KIF3B-knockdown melanocytes showed reduced protein levels of TYRP1, TYR, PMEL and LAMP-2, and decreased fibril formation (Fig. 4A–C). In line with these results, the fluorescence staining of PMEL was dramatically reduced in the peripheral cytosol (arrows) and a pool was mislocalized to lysosomes (arrowheads) [*r*=0.05±0.01in KIF3A sh-1; 0.05±0.02 in KIF3B sh-1 (both *P*≤0.01); compared to 0.14±0.02 in control cells] (Fig. 4A). Moreover, the formation of Mβ from total PMEL was not affected in KIF3A or KIF3B shRNA cells (Fig. 4B), similar to in Rab4A-depleted melanocytes (Fig. 1H). Additionally, LAMP-2- or EEA1-positive structures were clustered near the perinuclear region (arrows) upon KIF3A or KIF3B-knockdown in melanocytes, indicating that KIF3 regulates the positioning of these organelles (Fig. 4A) and melanosome biogenesis in a similar manner to Rab4A.

Next, we examined the KIF3 interaction with Rab4A, AP-3 and rabenosyn-5 and other effectors in HeLa cells owing to low plasmid transfection efficiency of melanocytes. Surprisingly, Myc–KIF3A showed strong interaction with the Rab4A, AP-3 (δ subunit) and rabenosyn-5, but not with Rabip4/4′ (Fig. 4D). We predict that these molecular interactions also exist in melanocytes. In line with this hypothesis, the localization of AP-3 near the perinuclear region was drastically reduced in KIF3A- and KIF3B-depleted melanocytes (data not shown). In addition, the total rabenosyn-5 level was unaffected upon KIF3A or KIF3B depletion in WT melanocytes (Fig. 4B). These results suggest that KIF3 associates with Rab4A–rabenosyn-5–AP-3, in which Rab4A possibly mediates the assembly of this complex.

### Rab4A regulates endosomal localization of KIF3 and AP-3 and controls cargo segregation on SEs

**Fig. 5.**
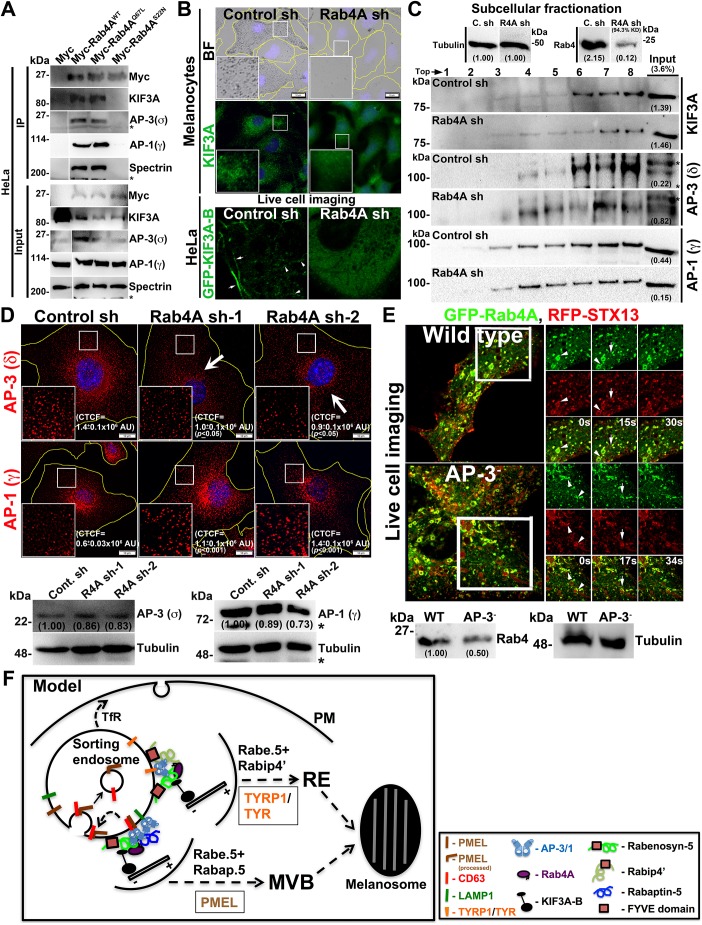
**Rab4A regulates the recruitment and association of KIF3 and AP-3 to endosomal membranes, and model illustrating Rab4A function in sorting cargo on SEs.** (A) Immunoprecipitation of Myc–Rab4A (WT, Q67L and S22N mutants) in HeLa cells. Both input and IP blots were probed as indicated. Spectrin was used as positive control for IP. (B,D) BF, IFM and live-cell imaging of Rab4A-knockdown cells. Arrowheads point to the KIF3 localization in HeLa cells. Arrows show the loss in AP-3 staining. Nuclei are stained with Hoechst 33258. The insets are a magnified view of the white boxed areas. The CTCF values are indicated separately (mean±s.e.m.). Scale bars: 10 µm. (C) Subcellular fractionation of control and Rab4A sh melanocytes, probing the cell fractions for localization of KIF3A, AP-3 and AP-1. (D,E) Immunoblotting analysis of adaptor subunits and Rab4 in respective cell types as indicated. Tubulin was used as a loading control. *non-specific bands. Protein band intensities were quantified and are indicated on the gels. (E) Super-resolution live cell imaging of GFP–Rab4A with respect to RFP–STX13 in wild-type and AP-3^−^ melanocytes. Arrows and arrowheads point to the localization of proteins to the REs and vesicles arising from VEs or SEs, respectively. The insets are a magnified view of the white boxed areas at indicated time points (Movies 1 and 2). (F) Proposed model wherein TYRP1 and TYR are segregated at the rabenosyn-5–Rabip4′ domains, and PMEL, CD63 and LAMP-1 are segregated at the rabenosyn-5–rabaptin-5 domains. In both these domains, cargo tails bind to the AP-3, which associates with Rab4A and KIF3 motor for positioning the domains. After segregation, TYRP1 and TYR enter into the REs for targeting toward maturing melanosomes, whereas PMEL is proteolytically cleaved and internalized into the ILVs of MVB along with CD63 (but not LAMP-1) for the biogenesis of stage II melanosomes.

We examined whether the interaction of Rab4A with KIF3 and AP-3 is dependent on the nucleotide status of Rab4A. Immunoprecipitation of ectopically expressed Myc-tagged Rab4A^WT^ and Rab4A^Q67L^ (a constitutively active mutant), but not Rab4A^S22N^ (a dominant-negative mutant), revealed a strong interaction with both KIF3A and AP-3 (σ), indicating that GTPase cycle of Rab4A is required for their interaction. Interestingly, we also observed an interaction between Rab4A and AP-1 (used as a negative control) in the HeLa cell lysates (Fig. 5A), which suggests that Rab4A independently interacts with these adaptor complexes (see below). Additionally, we predict that these molecular interactions exist in melanocytes. Next, we studied whether the recruitment of KIF3 and AP-3 on endosomal membranes is dependent on Rab4A. IFM analysis showed that KIF3A localized as punctate structures and appeared as diffused cytosolic staining in WT and Rab4A-depleted melanocytes, respectively (Fig. 5B). Owing to the difficulty in transfecting KIF3A/B constructs into melanocytes, we tested the motor localization in HeLa cells. Live-cell imaging analysis showed that GFP-KIF3A/B (co-expressed with GFP-KIF3A and GFP-KIF3B) localized as both punctate structures resembling endosomes and long tubular structures, which is a hallmark of recycling endosomes in HeLa cells (Delevoye et al., 2014) (Fig. 5B, shown is one frame of a movie, movie is not shown). As predicted, GFP–KIF3A/B completely localized to the cytosol in Rab4A-knockdown HeLa cells (Fig. 5B). Consistent with this result, subcellular fractionation showed that localization of KIF3A to endosomal membranes (fractions 6–8) was notably reduced upon Rab4A-knockdown compared to what was seen in WT melanocytes (Fig. 5C). These studies indicate that Rab4A recruits KIF3 onto endosomal membranes.

In contrast to KIF3, the IFM intensity of AP-3 (stained for the δ subunit), but not AP-1 (stained for the γ subunit), was significantly reduced in Rab4A-depleted compared to control melanocytes [CTCF for AP-3=1.0±0.1×10^6^ AU in Rab4A sh-1, 0.9±0.1×10^6^ AU in Rab4A sh-2 (both *P*≤0.05) compared to 1.4±0.1×10^6^ AU in control cells; CTCF for AP-1=1.1±0.1×10^6^ AU in Rab4A sh-1, 1.4±0.1×10^6^ AU in Rab4A sh-2 (both *P*≤0.001) compared to 0.6±0.03×10^6^ AU in control cells] (Fig. 5D). However, the total AP-3 or AP-1 subunit protein levels were not affected in Rab4A-knockdown melanocytes (Fig. 5D), suggesting that Rab4A either regulates the recruitment of AP-3, but not AP-1, onto endosomal membranes or is required for AP-3 stability on those membranes. Consistent with the first hypothesis, subcellular fractionation analysis showed that membrane-bound AP-3 levels, but not AP-1 levels, were reduced, and the localized AP-3 was distributed to the multiple organelle membranes (data not shown) in Rab4A-depleted but not in control melanocytes (Fig. 5C). Similarly, rabenosyn-5 knockdown in melanocytes also reduced the perinuclear distribution of AP-3 (data not shown). These studies suggest that Rab4A–rabenosyn-5 partly regulates the recruitment/association of AP-3 onto selective endosomal membranes.

We examined whether AP-3 plays a role in regulating the localization or activity of Rab4A in melanocytes. Previous studies have shown that Rab4A localizes to early/recycling tubular structures in fibroblasts (D'Souza et al., 2014; Sönnichsen et al., 2000; Van Der Sluijs et al., 1991). Super-resolution live-cell imaging of WT melanocytes showed that GFP–Rab4A localized as enlarged vacuolar ring-like structures with emanating vesicles positive for RFP–STX13 (Movie 1 and Fig. 5E, arrowheads), possibly representing EEs/SEs (Jani et al., 2015; Setty et al., 2007). Additionally, a large population of GFP–Rab4A also appeared as punctate structures positive for STX13 and emanating tubular structures positive for only STX13 (Movie 1 and Fig. 5E, arrows), likely corresponding to SEs/REs. Consistent with this, Rab4A fractionated into multiple subcellular fractions that correspond to EEs/REs (Fig. S4A). By contrast, GFP–Rab4A in AP-3^−^ melanocytes localized to both STX13-positive enlarged vacuolar structures (Fig. 5E arrowheads), which is similar to its localization in WT cells, and to longer tubular structures (Movie 2 and Fig. 5E, arrows), representing the EEs/SEs and REs, respectively (Dennis et al., 2015). Although the Rab4 level was slightly reduced, its localization to the recycling tubular endosomes was moderately increased in AP-3-deficient melanocytes compared to WT melanocytes (Fig. 5E). Thus, these studies in melanocytes demonstrate that Rab4A predominantly localizes to SEs and partly to EEs/REs, and its recruitment is independent of AP-3.

We further tested whether Rab4A-regulated cargoes are sorting substrates of AP-3 and/or AP-1 complexes. Amino acid sequence analysis of the cargoes showed that PMEL and TYR contain a (D/E)xxxL(L/I) motif and that CD63 and LAMP-1 contain a Yxxφ motif in the C-terminus (Fig. S4D) (Bonifacino and Traub, 2003). Studies have shown that AP-3 regulates the sorting of TYR (Theos et al., 2005), CD63 (Rous et al., 2002) and LAMP-1 (Dell'Angelica et al., 1999; Peden et al., 2004) proteins on endosomal membranes. However, the role of AP-3 and/or AP-1 in regulating the trafficking of PMEL remains unclear, although the C-terminus PMEL contains a putative acidic dileucine motif. In contrast, studies have shown that the dileucine motif of PMEL interacts with AP-2 during its internalization from the cell surface (Valencia et al., 2006). Here, we examined the interaction of Rab4A-dependent cargo tails with AP-3 or AP-1 subunits by means of a yeast two-hybrid (Y2H) assay (Fig. S4D). Similar to what was found in earlier studies, the dileucine motif of PMEL (in a longer C-terminal tail than previously studied) did not interact with either the AP-3 or AP-1 subunits (Fig. S4D). However, the tyrosine-based motif of CD63 showed strong interaction with AP-3 (the µ subunit) but not with AP-1 subunits. We predict that PMEL trafficking in melanocytes is possibly regulated through CD63 (van Niel et al., 2011). Consistent with this, AP-3-deficient melanocytes mislocalize both PMEL and GFP–CD63 to LAMP-2-positive compartments (Fig. S4E), similar to what is seen in Rab4A-knockdown cells (Fig. S1O). In line with these results, AP-3-deficient cells showed defective PMEL processing and fibril formation (Fig. S4E). However, PMEL was not segregated into exosomes (Fig. S4E) in AP-3-deficient cells, in contrast to Rab4A-depleted melanocytes (Fig. S1N), suggesting that PMEL is directly targeted to lysosomes for degradation upon loss in expression of AP-3 subunits. Thus, these data indicate that both AP-3 and Rab4A are required for sorting of PMEL as well as CD63 on SEs. As expected, LAMP-1 showed a strong interaction with the µ subunit of both AP-3 and AP-1 (Fig. S4D). Overall, these studies suggest that the majority of Rab4A-dependent cargoes are sorted by the AP-3 complex on endosomal membranes.

## DISCUSSION

SEs are the central regulatory hubs for targeting cargo either to the cell surface through recycling or to lysosomes for degradation. These organelles originate from EEs and mature into REs/LEs on post cargo sorting. Interestingly, in melanocytes, a few of these pathways are diverted towards the biogenesis of pigment granules. Within these pathways, the structural and enzymatic proteins of melanosome follow three independent transport routes: BLOC-1-mediated TYRP1 and ATP7A transport, AP-3-dependent TYR transport, and CD63-dependent PMEL transport to melanosomes. However, the specific cargo segregation mechanisms on SEs are unknown. Moreover, these processes are essential for both proper trafficking of cargo to the target organelle and organelle homeostasis.

In general, Rab GTPases function in membrane identity, cargo sorting and membrane fusion processes (Ohbayashi and Fukuda, 2012; Stenmark, 2009; Zerial and McBride, 2001). These GTPases recruit specific effector proteins onto the membranes and form transient local subdomains that are involved in cargo segregation, packaging and vesicle/tubule generation, which are then delivered towards the target membranes (Gruenberg, 2001). We hypothesized that similar subdomains exist on SEs for the segregation of melanosome-specific and general cargo in melanocytes. In search for a Rab regulator of melanosome cargo segregation, our RNAi screen identified Rab4A, which led to the mislocalization of all primary melanocytic cargoes to lysosomes upon its depletion. In contrast, Rab4A in fibroblasts has been shown to regulate the fast recycling of TfR (van der Sluijs et al., 1992) from EEs or GLUT4 vesicles (Aledo et al., 1995) to the plasma membrane. Furthermore, Rab4A has been shown to localize to the transient compartments of EEs and REs (Sönnichsen et al., 2000; Van Der Sluijs et al., 1991), and also orchestrates the endosomes by interacting with AP-1 or AP-3 adaptors via the Arf1-ARL1-dependent GTPase cascade (D'Souza et al., 2014). Nevertheless, the function of Rab4A in cargo segregation/transport pathways during melanosome biogenesis remains elusive. In this study, we extensively characterized the function of Rab4A in segregating and organizing the cargo into different subdomains, followed by its role in trafficking of cargo to the maturing melanosomes. During this process, Rab4A associates with rabenosyn-5 on endosomes and interacts with AP-3 on one side and KIF3 motor on other side (Fig. 5F). These interactions form a novel endosomal complex that further associates with either Rabip4/4′ or rabaptin-5 molecules, which possibly facilitate the melanosomal and lysosomal cargo segregation on SEs. This segregation was found to be essential for the targeting of cargo to REs and for the maturation of late endosomes or premelanosomes, in the case of melanocytes. Thus, Rab4A acts as a key organizer of endosomal domains during segregation and trafficking of multiple cargoes on SEs. This endosomal domain organization possibly either generates cargo-specific membrane domains on SEs or regulates the positioning/distribution of specific cargo-containing endosomes. We favor the first model because: (1) Rab4A depletion increases the accumulation of vacuolar endosomes compared to the control cells; (2) Rab4A knockdown increases the secretion of proteolytically unprocessed PMEL into exosomes, and mislocalizes both TYRP1 and TYR to lysosomes; (3) Rab4A inactivation alters the trafficking of the non-melanocytic cargoes CD63 and LAMP1/2; (4) Rab4A associates with AP-3, rabenosyn-5 and KIF3 (referred to here as the Rab4A complex) on endosomes, as evident by subcellular fractionation; (5) Rab4A-, rabenosyn-5- and KIF3-specific depletions in melanocytes phenocopy the hypopigmentation and defective fibril formation phenotypes; (6) rabenosyn-5, rabaptin-5 and Rabip4′ independently get recruited and moderately regulate the expression/endosomal localization of each other, but the association of rabenosyn-5 with Rabip4/4′ or rabaptin-5 distinguishes the cargo specificity during the segregation; and (7) finally, Rab4A knockdown phenotypes are specific and cannot be attributed to any change in either the transcription profile or Rab5A-localization/recruitment to the membranes. Thus, these results strongly support the hypothesis that Rab4A acts as a master regulator in segregating melanosomal and lysosomal cargo on SEs following individual transport to premelanosomes or lysosomes.

Our studies demonstrate that Rab4A regulates the formation of subdomains on SEs by interacting with Rab5A effectors and the cargo-sorting adaptor AP-3 (Fig. 3D). Moreover, we predict that Rab4A coordinates these molecules through its transient interactions on the endosomal membranes, where rabenosyn-5 and AP-3 (but not KIF3) are recruited independently of Rab4A. Additionally, rabenosyn-5 further recruits or associates with either Rabip4/4′ or rabaptin-5 and stabilizes Rab4A complex (Fig. S3). This model is consistent with our results showing that depletion of either Rabip4 or rabaptin-5 partially mimics the cargo trafficking defects observed in rabenosyn-5- or Rab4A-knockdown melanocytes. Thus, the Rab4A-Rab5A-shared effectors are very likely to act as adaptors on endosomal membranes to assemble the complex, which is mediated through Rab4A. Consistent with this proposal, Rab4A depletion reduced the association of AP-3 on the endosomal membranes, dissociated the KIF3 motor and dispersed the enlarged rabenosyn-5 or Rabip4′-positive endosomes to the periphery (Fig.5; Fig. S3). Several individual studies also support these interactions: (1) Rabip4 has been shown to interact with AP-3 (β subunit) and regulate endosomal cargo recycling and the distribution of lysosomes (Fouraux et al., 2004; Ivan et al., 2012) in fibroblasts; (2) rabaptin-5 has been shown to interact with AP-1 (γ subunit) and regulate Tf recycling (Deneka et al., 2003); similarly, rabenosyn-5 regulates TfR recycling (Navaroli et al., 2012); (3) Rab4A has been shown to associate with AP-1 or AP-3 localized domains on endosomes (D'Souza et al., 2014); (4) Rab4A has been shown to localize to the KIF3A-enriched membrane fractions (Bananis et al., 2004); and (5) Rab4–KIF3 has been shown to mediate insulin-induced GLUT4 exocytosis (Imamura et al., 2003). Although these interactions were observed primarily in fibroblasts, none of these studies integrated their role in other cargo transport pathways. To our knowledge, this study is the first to show the specificity of these interactions in selective cargo transport to melanosomes. However, our study did not illustrate the role of the following known interactions in melanosome biogenesis, which need to be evaluated in future: (1) rabenosyn-5 has been shown to interact with VPS45 and EEA1 (Nielsen et al., 2000); (2) rabaptin-5 has been shown to interact with Rab4 and AP-1 (γ subunit) (Deneka et al., 2003; Pagano et al., 2004); (3) Rab4A-GTP has been shown to recruit various effectors such as GRASP1 (Hoogenraad et al., 2010), D-AKAP2 (Eggers et al., 2009), Gadkin (Schmidt et al., 2009) and RCP (Lindsay et al., 2002); and (4) KIF3A/B has been shown to interact with KAP3 (Yamazaki et al., 1996). Here, we predict that Rab4A or Rab4A-Rab5A-shared effectors might have additional roles in regulating trafficking steps other than those involved in transporting melanosomal and lysosomal cargoes.

Our studies show that Rab4A acts as a key regulator in the cargo trafficking to melanosomes. We demonstrated that Rab4A regulates the sorting of PMEL (through CD63) and LAMP-1, through its interaction with AP-3. Here, we hypothesized that PMEL–CD63 and LAMP-1-bound AP-3 associate with Rab4A-rabenosyn-5–rabaptin-5–KIF3 molecules on SEs and generate a distinct subdomain that directs cargo towards LEs (Fig. 5F). Inactivation of Rab4A reduces the membrane association of AP-3, which results in the internalization of unproteolyzed PMEL–CD63 into the ILVs, which is then secreted as exosomes, resulting in a reduced number of stage II melanosomes (Fig. S1N; Fig. 2). In contrast, Rab4A possibly segregates both TYRP1 and TYR on SEs by interacting with AP-3, which further associates with rabenosyn-5–Rabip4/4′–KIF3 and generates a different subdomain that guides the cargo towards REs (Fig. 5F). Upon depletion of Rab4A, both TYRP1 and TYR enter into the classical ubiquitin-dependent lysosomal degradation pathway, and a pool recycles back to the cell surface (Fig. 1; Fig. S1M,Q). Moreover, this model further supports the BLOC-1-dependent TYRP1 trafficking to the melanosome, which occurs on REs (Delevoye et al., 2009). Thus, Rab4A acts a master regulator of cargo segregation by generating different subdomains through its association with combinations of Rab4A-Rab5A-shared effectors on SE membranes.

## MATERIALS AND METHODS

### Reagents and antibodies

All chemicals and reagents were purchased either from Sigma-Aldrich (Merck) or ThermoFisher Scientific (Invitrogen). Puromycin from Calbiochem and Matrigel from BD Biosciences were purchased. Commercial antibodies with their specific use (IB, immunoblotting; IFM, immunofluorescence microscopy; IP, immunoprecipitation and FACS, fluorescence-activated cell sorting) and the catalog numbers are as indicated. Antibodies against KIF3A (IF, IB; ab11259), LIMPII (IB; ab16522) and PMEL (HMB45; IF, IB; detects pre-melanosomes in IF and detects fibrils on IB; ab787) were from Abcam; TYRP1 (TA99; IF, FACS; HB-8704) was from American Type Culture Collection; γ-adaptin (AP-1; IF, IB; 610385), LAMP1 (IB; 553792), Rab4 (IB, IF, IP; 610889; specific to Rab4A and low affinity against mouse Rab4), rabaptin-5 (IB, IP; 610676) and TfR (CD71; FACS; 553264) were from BD Biosciences; alpha II-spectrin (SPTAN1; IB; A301-249A) was from Bethyl; EEA1 (IF; 3288), HSP90 (IB; 4877), LC3A/B (IF; 4108), Rab5 (IF, IB; 3547) and Rab11 (IB, 5589) were from Cell Signaling Technology; δ-adaptin (AP-3, IF; SA4), LAMP-1 (IB, FACS; 1D4B) and LAMP-2 (IF, IB; GL2A7) were from Developmental Studies Hybridoma Bank; GFP (IB; A11122) was from Invitrogen; γ-tubulin (GTU88; IB; T6557) was from Sigma-Aldrich; σ3-adaptin (AP-3; IB; sc-136338), GAPDH (IB; sc-25778), c-Myc (IB; sc-789), TfR (CD71; IB; sc-7087) and TYRP1 (IB; sc-25543) were from Santa Cruz Biotechnology. All secondary antibodies were either from Invitrogen or Jackson Immunoresearch. Antisera to Rabip4/4′ (IB) (Ivan et al., 2012), STX13 (IF, IB) (Prekeris et al., 1998) and TYR (PEP7h; IF, IB) (Theos et al., 2005) have been described previously. Other antisera such as δ-adaptin (dh2, AP-3; IB) (Andrew Peden, University of Sheffield, Sheffield, UK); anti-PmelN (N-terminus to PMEL; IB) and anti-Pep13h (PMEL-C, C-terminus to PMEL; IB; used for PMEL processing) (Michael S. Marks, University of Pennsylvania, Philadelphia, USA) and rabenosyn-5 (Silvia Corvera, UMASS Medical School, Worcester, USA) were obtained as gift from respective laboratories mentioned in the parenthesis.

### Plasmids and shRNAs

#### Expression constructs

Myc-Rab4A^WT^: Full-length human Rab4A was PCR amplified with an N-terminal Myc epitope sequence from human cDNA and subcloned into the BamH1 and XhoI sites of pCDNA3.1(+) (Invitrogen). GFP-Rab4A^WT^: PCR amplified full-length human Rab4A (without a Myc tag) was digested with EcoRI and XhoI enzymes and subcloned into the EcoRI and SalI sites of pEGFP-C2 (Clontech). GFP-Rab4A^sh2R^: Mutagenesis of Rab4A^WT^ DNA sequence at 436–459 bases (changed at wobble base of amino acid sequence QENELMFL; 5′-CAAGAAAATGAGCTGATGTTTTTG-3′ converted into 5′-CAGGAAAACGAATTAATGTTTTTG-3′) was carried out in pEGFP-C2-Rab4A^WT^ plasmid using a QuikChange multi site-directed mutagenesis kit (Agilent Technology). Note, this plasmid is resistant to the Rab4A shRNA-2. Myc–Rab4A^Q67L^ and Myc-Rab4A^S22N^: Mutagenesis of the glutamine residue at the position 67 to a leucine residue, and the serine residue at position 22 to an asparagine residue separately in pCDNA3.1-Myc-Rab4A^WT^ was carried out using the QuikChange multi site-directed mutagenesis kit. Empty vector pCMV-Myc was from Clontech. GFP–CD63 (62964, deposited by Paul Luzio) and GFP–rabenosyn-5 (37538, deposited by Silvia Corvera; Navaroli et al., 2012) were obtained from Addgene. GFP–STX13 and RFP–STX13 (Jani et al., 2015); Myc–KIF3A (Azevedo et al., 2009); pCI-Pmel17 (referred to here as PMEL) (Berson et al., 2001) have been described previously or were kind gift from their respective laboratories. GFP–KIF3A and GFP-KIF3B constructs were obtained from Alistair Hume (with the permission from Tetsu Akiyama, Japan), University of Nottingham Medical School, Nottingham, UK (Haraguchi et al., 2006). GFP–Rabip4′ has been described in Ivan et al. (2012), and mCherry–rabaptin-5 and CFP–rabaptin-5 were subcloned from pCDNA3-rabaptin-5 (Nagelkerken et al., 2000).

#### TRC shRNA vectors

We selected human shRNA plasmids encoding target sequence against the multiple Rab proteins that are highly conserved for mouse Rab GTPases. These shRNAs were purchased from TRC Genome-wide shRNA library (Sigma-Aldrich). The target sequence and their percentage similarity with mouse proteins are listed in Table S1.

#### Retroviral shRNA vectors

Oligodeoxyribonucleotide duplexes containing the target sequences (listed in Table S2) were cloned into the BamH1 and HindIII sites of pRS shRNA vector (OriGene Technologies). Empty pRS shRNA plasmid was used as a control in all shRNA knockdown experiments. Rabip4 shRNAs also target the longer Rabip4′ isoform in the WT melanocytes.

#### Yeast-two hybrid vectors

Empty vectors and the plasmids for Y2H, containing different subunits of AP-3 (δ, µ3, β3A, β3A-hinge and σ3) or AP-1 (γ, µ1 and σ1), were as described previously (Jani et al., 2015). Oligodeoxyribonucleotide duplexes corresponds to C-terminal tails of hPMEL^623-668^, hCD63^227-238^, hLAMP-1^406-417^ and mTYR^502-533^ were cloned into the EcoRI and SalI sites of pGBKT7. All plasmid inserts were verified by DNA sequencing.

### Yeast two-hybrid assay

The detailed protocol of the Y2H assay are as described in Jani et al. (2015). Briefly, the Y2HGold yeast strain (Clontech) was transformed with different bait and prey plasmids as indicated in the figure (Table S4) by a lithium acetate transformation protocol. The yeast transformants were selected on minimal medium plates supplemented with complete amino acid mix (Y0750, Sigma-Aldrich), lacking leucine and tryptophan (referred to here as +His medium). Next, transformants were grown to exponential phase, serially diluted and then spotted on +His, −His (Y2146 –Sigma-Aldrich) and −His [+2 or 10 mM 3AT (3-Amino-1,2,4-triazole)] plates. Plates were incubated for 3–5 days at 30°C and then imaged under white light in a Bio-Rad Molecular Imager. The yeast transformants that grew on –His (+3AT) were considered as demonstrating a positive interaction between bait and prey proteins.

### Cell culture, transfection and retroviral transduction

The following immortal mouse melanocyte cell lines were used in this study. Wild-type melan-Ink4a is derived from C57BL/6J *a*/*a Ink4a*-*Arf^−/−^* mice, formerly called melan-Ink4a-1 and referred to here as WT or melan-Ink4a (Ha et al., 2007). AP-3^−^ melan-mh is derived from C57BL/6J *Ap3d^mh/mh^* mice and referred to here as AP-3^−^ or melan-mh (Jani et al., 2015). Cells were maintained as described previously (Jani et al., 2015). DNA vectors were transfected into the melanocytes or PLAT-E cells (Cell Biolabs) or HeLa cells (ATCC) by using Lipofectamine 2000 (Invitrogen) according to the manufacturer's protocol. For gene knockdown, wild-type melanocytes were transduced with retroviruses encoding different target sequences in pRS shRNA plasmids, isolated from PLAT-E cells (Morita et al., 2000). Melanocytes were selected twice with puromycin (2 μg/ml) on the second and fifth day of retrovirus transduction. In some experiments, control/shRNA knockdown cells or AP-3^−^ melanocytes were transfected with GFP–CD63, GFP–rabenosyn-5, GFP–Rabip4′ or mCherry–rabaptin-5 separately or with a mixture of GFP–KIF3A and GFP–KIF3B or GFP–Rab4A and RFP–STX13. In one experiment, HeLa cells were directly transfected with Rab4A shRNA (as listed in Table S1) using Lipofectamine 2000 and selected the cells twice with puromycin (2 μg/ml) on the second and fifth day.

### Transcript analysis by semiquantitative PCR

Melanocytes grown in a 60 mm dish were treated with Trizol reagent (Sigma-Aldrich) and then extracted with chloroform at room temperature. Further, the aqueous layer was precipitated with isopropanol followed by a wash with 70% ethanol. Finally, the isolated RNA pellet was air dried and suspended in 0.01% DEPC treated water (Sigma-Aldrich). The cDNA was prepared by using a cDNA synthesis kit (Fermentas) after estimating the RNA concentration using NanoDrop 2000C spectrophotometer (Thermo Scientific). Gene transcript levels were analyzed by semiquantitative PCR (Bio-Rad S1000 Thermal Cycler) using gene specific primers (listed in Table S3) and an equal amount of cDNA from each sample. In all PCRs, GAPDH was used as a loading control. Band intensities were measured, normalized to that of GAPDH, and the fold change with respect to the control quantified and then listed in the figure.

### Melanin estimation

The intracellular melanin content of melanocytes was measured using a protocol as described previously (Mahanty et al., 2016). Cells were transfected with respective shRNAs or transduced with virus encoding control or different Rab4A shRNAs. After puromycin selection, cells were harvested and lysed in lysis buffer [50 mM Tris-HCl pH 7.4, 2 mM EDTA, 150 mM NaCl, 1 mM DTT and 1× protease inhibitor cocktail (Sigma-Aldrich)] and then centrifuged for 15 min at 20,000 ***g*** and 4°C. Supernatants were subjected to protein estimation using a Bradford protein estimation kit (Bio-Rad). The melanin pellet fractions were washed with ethanol:diethyl ether (1:1 ratio), air dried and resuspended in a buffer containing 2 M NaOH and 20% DMSO followed by incubation at 60°C for 30 min. The optical density (OD) of melanin pigments were measured at 492 nm using a Tecan multi-well plate reader (Tecan) and then normalized to the respective protein concentration.

### Immunoblotting

Cell lysates were prepared using a protocol described previously (Setty et al., 2007) and γ-tubulin was used as a loading control in all experiments. Immunoblots were developed with the Clarity Western ECL substrate (Bio-Rad) and imaged in a Bio-Rad Molecular Imager ChemiDoc XRS+ imaging system equipped with a Supercooled (−30°C) CCD camera (Bio-Rad) using Image Lab 4.1 software. Protein band densities were measured, normalized to that of γ-tubulin, and the fold change with respect to control quantified and then indicated in the figure. The percentage Mβ formation was calculated from the total PMEL (sum of P1, P2 and Mβ band densities) after γ-tubulin normalization. In certain experiments, the percentage knockdown was also quantified after γ-tubulin normalization.

### Exosome preparation

Conditioned medium from subconfluent mouse melanocytes was collected every 48* *h and stored at 4°C before use. Initially, medium was cleared for cell debris by centrifuging consecutively at 2000 ***g*** and 4000 ***g*** for 15 min (4°C). The supernatant was further centrifuged at 10,000 ***g*** for 30 min (4°C) followed by a 100,000 ***g*** spin for 60 min (4°C) in a Ultracentrifuge (Beckman L-80) using 80 TI rotor. The exosome pellet was washed once with 1× PBS (pH 7.4), lysed in urea lysis buffer (8 mM urea, 50 mM Tris-HCl pH 7.4, 50 mM Na_2_HPO_4_, 300 mM NaCl, 0.5% NP40 and protease inhibitor cocktail) and then analyzed by immunoblotting.

### Extraction of melanosomal fibrils

Control and knockdown melanocytes were washed with 1× PBS and suspended in lysis buffer (20 mM Tris-HCl pH 7.4, 150 mM NaCl, 1 mM EDTA, 1% Triton X-100 and protease inhibitor cocktail) followed by lysing the cells using G25 syringe. Then, lysates were incubated on ice for 1* *h and then centrifuged for 10 min at 12,000 ***g*** (4°C). The pellets were suspended in lysis buffer containing 8M urea and then boiled for 30 min at 60°C. The suspensions were centrifuged (12,000 ***g*** for 10 min) and the supernatants collected, followed by analyzing via immunoblotting with the HMB45 antibody.

### *In vitro* tyrosinase activity and protease inhibitor assays

Cells on the Matrigel-coated coverslips were fixed and assayed for tyrosinase activity using the substrate L-DOPA as described previously (Atul Jani et al., 2016). Similarly, cells were treated with or without 50 nM bafilomycin A1 for 4* *h at 37°C before fixation, and then stained with respective antibodies followed by IFM (Jani et al., 2015). In some experiments, control and bafilomycin-treated cells were subjected to immunoblotting.

### Immunofluorescence microscopy and image analysis

For steady state localization studies, cells on coverslips were fixed with 2% formaldehyde (in PBS) and then stained with primary antibodies followed by the respective secondary antibodies as described previously (Setty et al., 2007). Bright field (BF) and immunofluorescence (IF) microscopy of cells was performed on an Olympus IX81 motorized inverted fluorescence microscope equipped with a CoolSNAP HQ2 (Photometrics) CCD camera using 60× (oil) U Plan super apochromat objective. Acquired images were deconvolved and analyzed using cellSens Dimension software (Olympus). Pigmentation (normal or hypopigmentation) in cells was quantified from BF images visually by counting ∼100 cells in each experiment. The average pigmentation in cells was calculated and then plotted. Similarly, a reduced fluorescence staining of TYRP1 or TYR in shRNA-depleted cells was quantified visually and then plotted as percentage of cells that had lost the staining of respective proteins (Fig. S1A). In Fig. S1F, melanocytes with ∼50 or fewer melanosomes/cell were considered as hypopigmented cells, counted visually from the randomly taken BF images of each condition and then plotted. The colocalization between two colors was measured by selecting equal square areas in the entire cell excluding the perinuclear area and then the Pearson's correlation coefficient (*r*) value was estimated using cellSens Dimension software. The average *r* value per each cell was calculated and then plotted or represented as mean value along with the s.e.m. Note that the maximum intensity projection of non-deconvolved *Z*-stack images were used for estimating the *r* values. The mean fluorescence intensity (MFI) of immunostained melanocytes was measured using ImageJ software and corrected total cell fluorescence (CTCF) was calculated using the below formula. CTCF (in arbitrary units, A.U. or AU)=area of the cell (MFI of cell−MFI of background). The averaged CTCF values from 10–25 cells/condition were calculated and indicated in the figure. The analyzed images were assembled using Adobe Photoshop.

### Live-cell imaging

Cells were plated on 2-cm glass-bottomed dishes (Mat Tek Corporation) and then transfected with respective constructs. After 24* *h, cells were visualized under an Olympus IX81 fluorescence microscope equipped with an environmental chamber maintained at 37°C with 5% CO_2_ and analyzed by cellSens Dimension software. Time lapse microscopy of both GFP and RFP was performed by capturing image streams over 3–5 min using a CoolSNAP HQ2 (Photometrics) CCD camera. Similarly, the time lapse imaging was performed on a Zeiss LSM880 laser scanning microscope with Airyscan mode using Zen lite 2.0 software to obtain the movies equivalent to the super resolution. Images were analyzed and converted into either TIFF or avi format for visualization.

### Electron microscopy

Control and Rab4A-knockdown melanocytes were seeded on Matrigel-coated glass coverslips. After 24* *h, cells were fixed initially with 0.5% Karnovsky's fixative (4% paraformaldehyde, 72 mM sodium cacodylate pH 7.4, 4 mM CaCl_2_, 0.5% glutaraldehyde) for 2* *h followed by overnight fixation with 2% Karnovsky's fixative (contains 2% glutaraldehyde). Cells were processed for Epon embedding as described previously (Raposo et al., 2001). Ultrathin sections of cell monolayers were prepared with a Reichert UltracutS ultramicrotome (Leica Microsystems) and contrasted with uranyl acetate and lead citrate as described previously (Raposo et al., 2001). Samples were examined with a FEI Tecnai Spirit electron microscope (FEI Company), and digital acquisitions were made with a numeric camera (Quemesa; Soft Imaging System). For quantification, melanosome stages were defined by morphology (Raposo et al., 2001) and vacuoles were defined as empty organelles. The number of melanosomes and vacuoles per µm^2^ cytosol were counted using ImageJ software. We counted ten cells from each control sh and Rab4A sh condition. Furthermore, we estimated the melanosome stages from 883 total melanosomes of control sh and 300 total melanosomes of Rab4A sh cells.

### Determining cell surface expression levels

Cells were harvested, washed with 1× PBS and then suspended in growth medium (supplemented with 25 mM HEPES pH 7.4) containing saturating concentrations of unconjugated primary antibodies on ice for 30–45 min. Cells were washed and incubated with respective Alexa Fluor 488-conjugated secondary antibody for 30–45 min on ice. Finally, cells were washed, suspended in ice-cold FACS buffer (5% FBS, 1 mM EDTA and 0.02% sodium azide in PBS) and the fluorescence intensity was measured using FACS Canto (BD biosciences). Data was analyzed using FlowJo (Tree Star) software and plotted as the mean fluorescence intensity (MFI) as described previously (Setty et al., 2007).

### Subcellular fractionation

Subcellular fractionation was carried out using a protocol described previously (Mahanty et al., 2016). Briefly, melanocytes were harvested, washed with 1× PBS and suspended in 0.25 M sucrose buffer (0.25 M sucrose, 1 mM EDTA, 25 mM HEPES pH 7.4, 0.02% sodium azide and protease inhibitor cocktail). Cells were homogenized on ice using a Dounce homogenizer and then clarified by centrifugation at 600 ***g*** for 10 min at 4°C. The cell lysate was fractionated on a sucrose step gradient (2.0 M, 1.6 M, 1.4 M and 1.2 M sucrose buffers manually layered from bottom to top in an ultracentrifuge tube) using a SW55Ti rotor by spinning at 160,000 ***g*** at 4^0^C for 4–6* *h in a Beckman L-80 ultracentrifuge. Fractions were manually separated and subjected to immunoblotting. In Fig. S4, the percentage enrichment of each protein in the fraction was calculated from the protein band densities, normalized to that in the first fraction and then plotted as a graph.

### Immunoprecipitation

HeLa cells expressing GFP alone (as a control) or GFP-tagged expression constructs were subjected to immunoprecipitation using GFP-Trap_A beads (Chromotek). Briefly, cells were lysed in lysis buffer (20 mM HEPES pH7.4, 100 mM KCl, 10 mM MgCl_2_, 5 mM EDTA, 1% Triton X-100, 100 µM GTPγS and protease inhibitor cocktail) on ice for 30 min. The lysates were centrifuged at 20,000 ***g*** for 10 min at 4°C and then incubated with equilibrated GFP-Trap_A beads for 4–5* *h under constant mixing at 4°C. The beads were washed twice with wash buffer (20 mM HEPES pH7.4, 100 mM KCl, 10 mM MgCl2, 5 mM EDTA and 0.1% Triton-100), suspended in 2× SDS-sample buffer and then analyzed by immunoblotting. Similarly, immunoprecipitation of Myc-tagged proteins expressing in HeLa cells or endogenous rabaptin-5 or Rab4 in melan-Ink cells was carried out using anti-Myc or anti-rabaptin-5 or anti-Rab4 antibodies, respectively. These lysates were incubated with Protein G–Sepharose 4B beads (Invitrogen) overnight under constant mixing at 4°C. Finally, beads were washed with wash buffer, suspended in sample buffer and analyzed by immunoblotting.

### Statistical analysis

Statistical significance was determined with an unpaired Student's *t*-test or via ANOVA using the GraphPad software. All values are given as the mean±s.e.m.

## Supplementary Material

Supplementary information
