# Supplementary material for: Rab4A organizes endosomal domains for sorting cargo to lysosome-related organelles
Source: J Cell Sci. 2018 Sep 20;131(18):jcs216226. doi: 10.1242/jcs.216226 (PMC6151265; doi:10.1242/jcs.216226)
Supplement: Supplementary information [file joces-131-216226-s1.pdf]

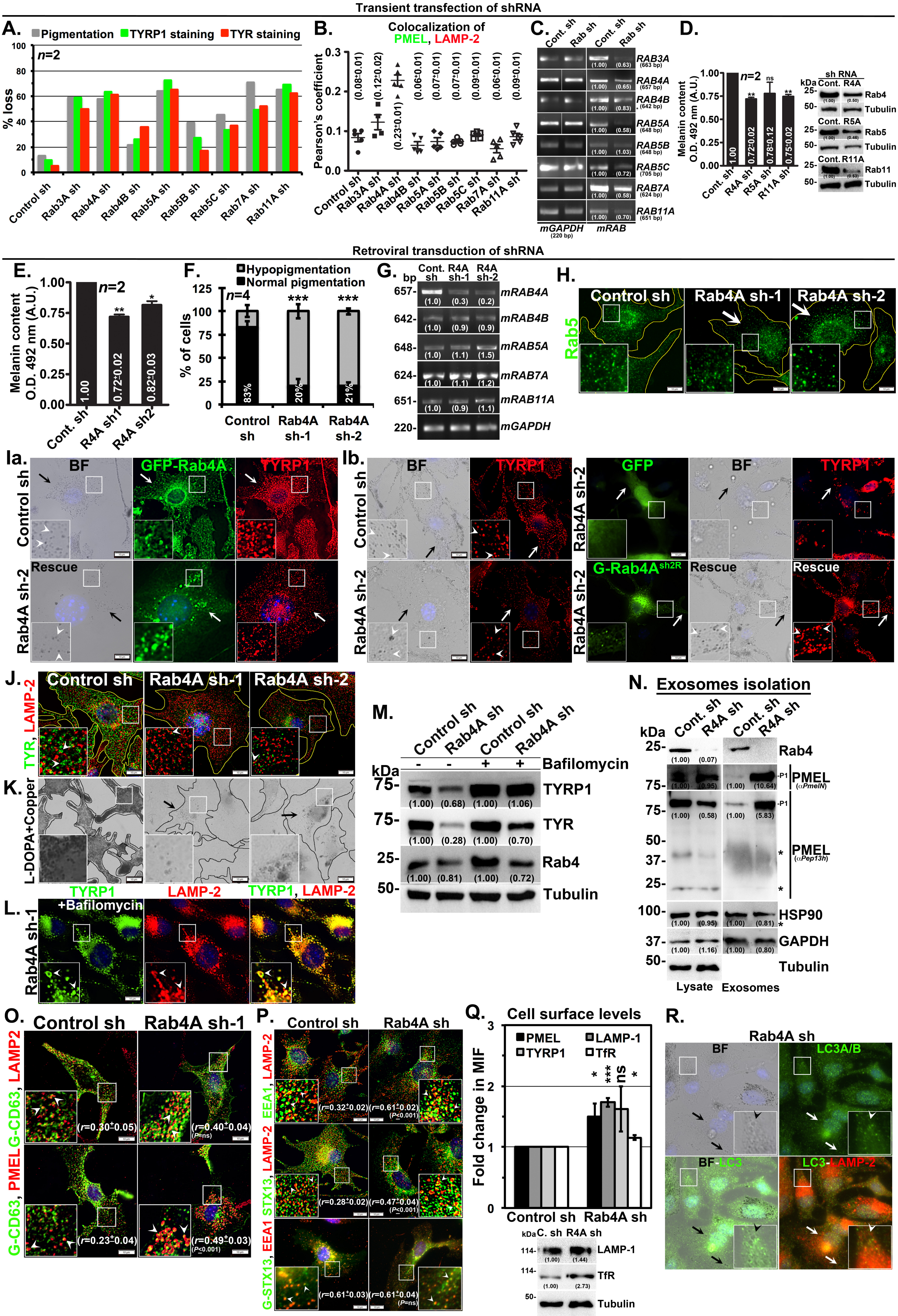

Journal of Cell Science • Supplementary information

Fig. S1. RNAi screening to identify the Rab GTPase that regulates cargo sorting on SEs. Rab4A-knockdown affects cargo trafficking and melanocyte pigmentation. (A-D) BF, IFM, transcript, immunoblotting and pigment analysis of wild-type melanocytes that were transiently transfected with control or Rab specific shRNAs as indicated. Cells were quantified for the respective phenotypes and then plotted (*n*=2; ≥100 cells/sample) (A). The colocalization efficiency (*r*) between PMEL and LAMP-2 in Rab-knockdown cells was measured and then plotted (B). Gene knockdown efficiency in the respective Rab-knockdown cells was measured using semiquantitative-PCR, quantified and indicated on the gels (C). Knockdown cell lysates were immunoblotted and subjected to pigment estimation separately (D). (E-R) Biochemical analysis of retroviral mediated Rab4A-knockdown in melanocytes. (E) Melanin content in the cells was measured, quantified and indicated (*n*=2). (F) Visual quantification of melanocyte pigmentation (*n*=4; ≥100 cells/sample). The percentage of normal pigmented cells was indicated separately. (G) cDNA from Rab4A-knockdown cells was analyzed for the expression of several endosomal Rabs as indicated. DNA band intensities were quantified and indicated on the gels. (H-L, Q, P, R) IFM and *in vitro*-TYR activity analysis of Rab4A-depleted melanocytes. Arrows indicate the loss in fluorescence staining of Rab5 (H) or rescue of melanocyte pigmentation/TYRP1 staining (Ia, Ib) or reduced TYR activity (K) or melanosome clusters (R). Arrowheads point to the pigmented melanosomes/TYRP1 (Ia, Ib) or colocalization between two proteins (J-L, O, P). The respective Pearson's coefficient (*r*) values were indicated separately (mean±s.e.m.). Nuclei are stained with Hoechst33258. The insets are a magnified view of the white boxed areas. Scale bars: 10 μm. Note, cells were transfected with GFP-Rab4A in Ia and Rab4A shRNA-2 resistant GFP-Rab4A (GFP-Rab4A^{sh2R}) in Ib. (M, N, Q) Analysis of proteins both in cell lysates and exosomes released by the control and Rab4A-knockdown cells. In M, cells were treated with bafilomycin. Tubulin and HSP90 or GAPDH were used as a loading control for cell lysates and exosomes respectively. P1, full length PMEL band. *, non-specific bands. Protein band intensities were quantified and indicated on the gels. (Q) Cell surface expression of proteins in Rab4A-knockdown melanocytes was measured using FACS and then plotted as fold change in mean intensity fluorescence (MIF). Statistical analysis includes *, $P \leq 0.05$; **, $P \leq 0.01$ and ***, $P \leq 0.001$ and ns, not significant.

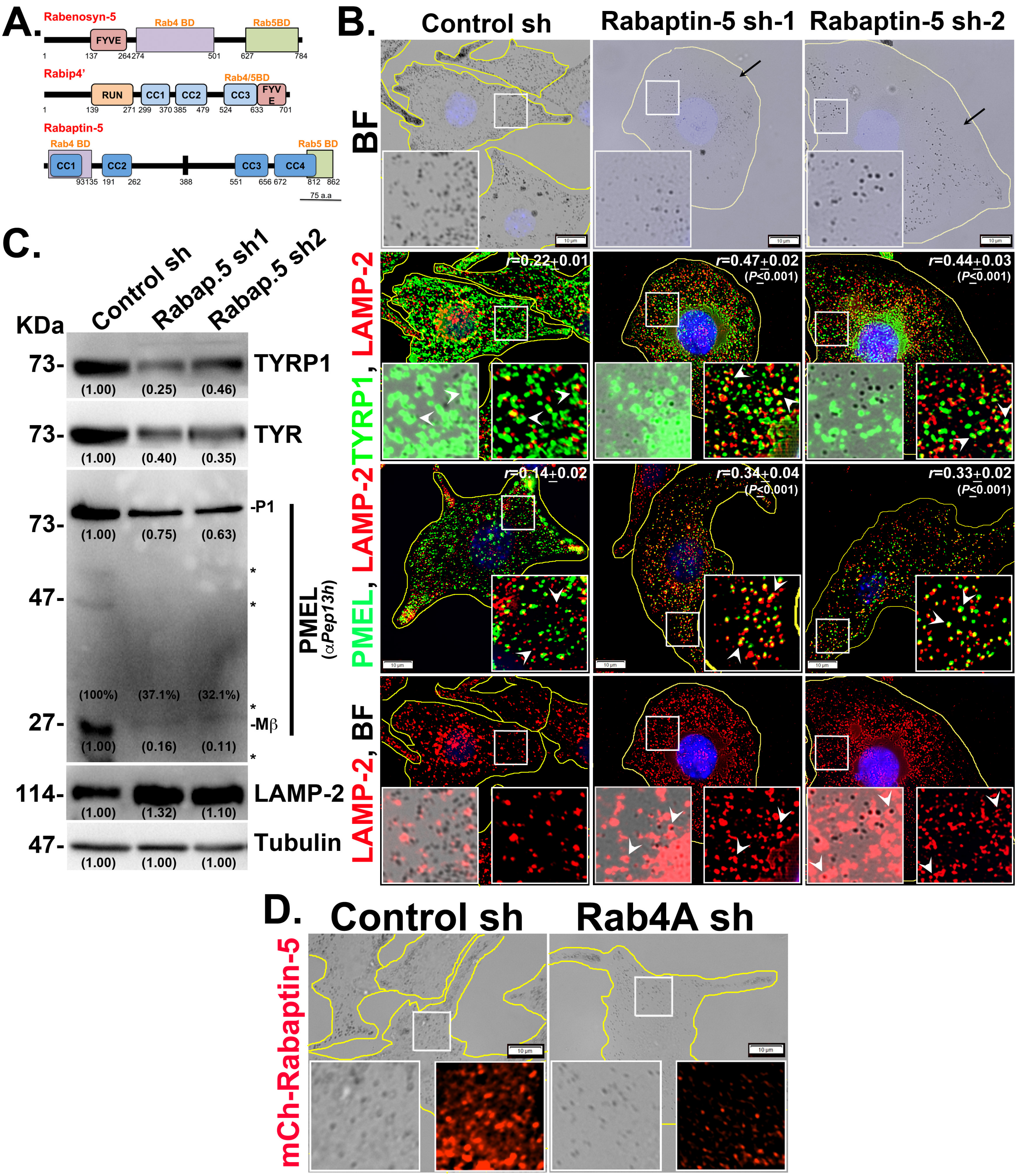

Journal of Cell Science • Supplementary information

Fig. S2. Rabaptin-5 regulates cargo sorting and melanocyte pigmentation. (A) Schematic diagram of conserved domains in three Rab4A-Rab5A-shared effectors. CC, coiled-coiled domain (blue); FYVE, PI3P binding domain (light red); RUN, Rab4 and Rab5 binding domains are shown separately. Rabip4 is the shorter isoform of Rabip4'. (B, D) BF and IFM analysis of Rabaptin-5-knockdown cells (sh-1 and sh-2) or mCherry-Rabaptin-5 expression in Rab4A-depleted melanocytes. Black arrows indicate the loss in pigmentation and arrowheads point to the cargo localization to lysosomes or melanosomes. The colocalization efficiency (*r*) between the proteins was indicated separately. Nuclei are stained with Hoechst33258. The insets are a magnified view of the white boxed areas. Scale bars: 10 μm. (C) Immunoblotting analysis of melanosomal and lysosomal proteins in knockdown cells and the tubulin used as a loading control. P1 and Mβ, full length and processed PMEL bands. *, non-specific bands. Protein band intensities were quantified and indicated on the gels.

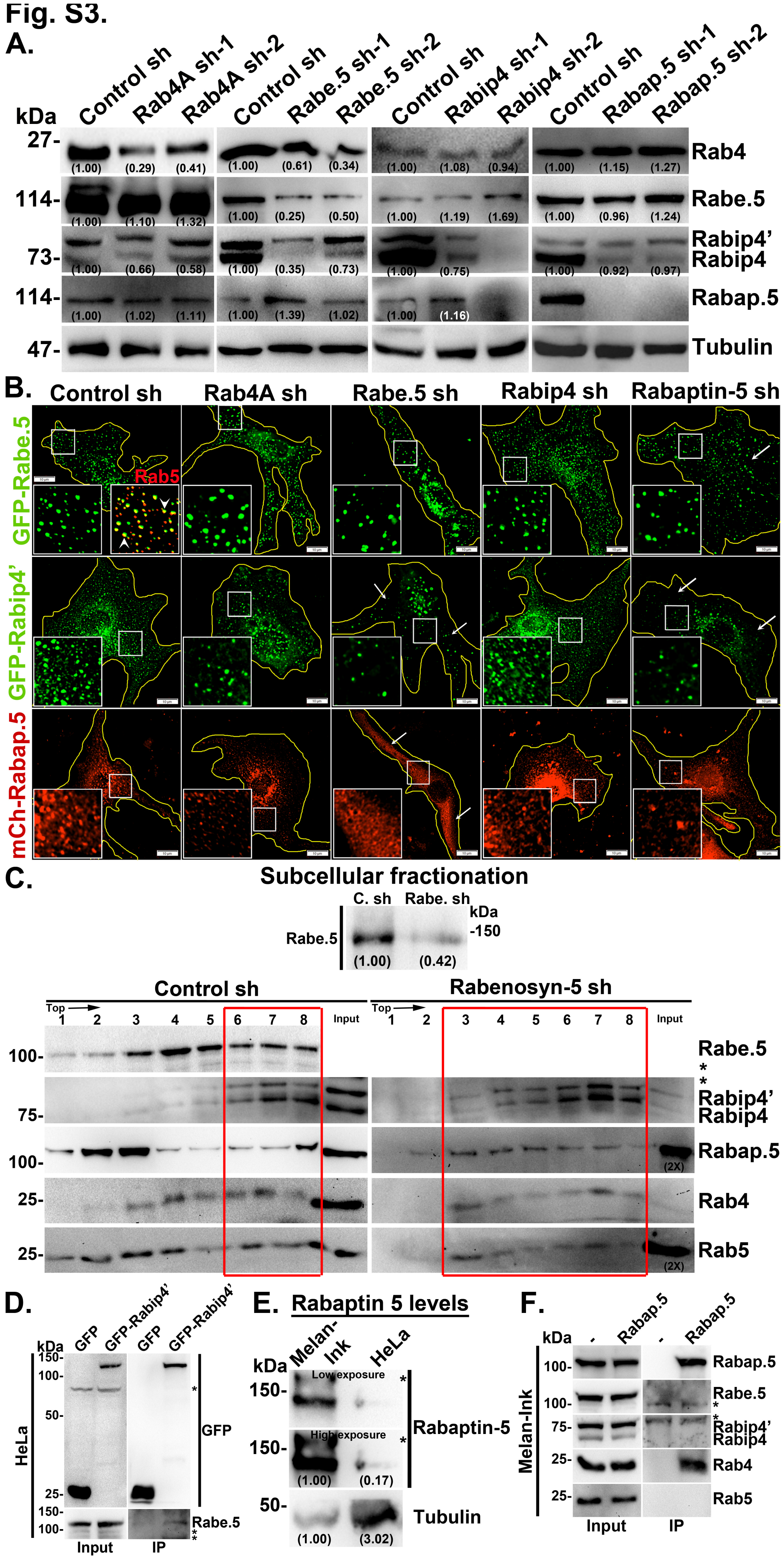
Fig. S3.
A.
Control sh
Rab4A sh-1
Rab4A sh-2
Rabe.5 sh-1
Rabe.5 sh-2
Rabip4 sh-1
Rabip4 sh-2
Rabap.5 sh-1
Rabap.5 sh-2
kDa
27-
114-
73-
114-
47-
Rab4
Rabe.5
Rabip4'
Rabip4
Rabap.5
Tubulin
B.
Control sh
Rab4A sh
Rabe.5 sh
Rabip4 sh
Rabaptin-5 sh
GFP-Rabe.5
GFP-Rabip4'
mCh-Rabap.5
Rab5
C.
Subcellular fractionation
C. sh
Rabe. sh
Rabe.5
-150
(1.00)
(0.42)
Control sh
Rabenosyn-5 sh
Top
Input
100-
75-
25-
Rab5
(2X)
D.
GFP
GFP-Rabip4'
150-
100-
50-
25-
HeLa
Input
IP
E.
Rabaptin 5 levels
Melan-Ink
HeLa
Low exposure
High exposure
Rabaptin-5
(1.00)
(0.17)
(3.02)
F.
Melan-Ink
-
Rab5

Fig. S3. Rab4-Rab5 shared effectors regulate each other stability and independently recruited to the endosomal membranes. (A, E) Immunoblotting analyses of Rab4A-Rab5A-shared effectors and Rab4 in Rab4A/Rabenosyn-5/Rabip4/Rabaptin-5 sh melanocytes. Similarly, total Rabaptin-5 protein levels were measured in both melan-Ink and HeLa cells. The low and high exposures of Rabaptin-5 probed gels were shown separately (E). Tubulin was used as a loading control. Protein band intensities were quantified and indicated on the gels. (B) IFM analysis of GFP/mCherry tagged Rab4-Rab5 shared effectors in Rab4A/Rabenosyn-5/Rabip4/Rabaptin-5 sh melanocytes. GFP-Rabenosyn-5 localizes to Rab5-positive endosomes in Control sh cells (inset, white arrowheads). Arrows indicate the decreased, dispersed or cytosolic localization of shared effectors in knockdown cells. The insets are a magnified view of the white boxed areas. Scale bars: 10 μm. (C) Subcellular fractionation of control and Rabenosyn-5 sh melanocytes and probed the fractions for localization of Rab4A-Rab5A-shared effectors, Rab4 and Rab5. Red box indicates the distribution of the molecules in different fractions. (D, F) Immunoprecipitation of GFP-Rabip4' in HeLa cells or endogenous Rabaptin-5 in wild-type melanocytes. Both cell lysate (input) and IP blots were probed as indicated. *, non-specific bands.

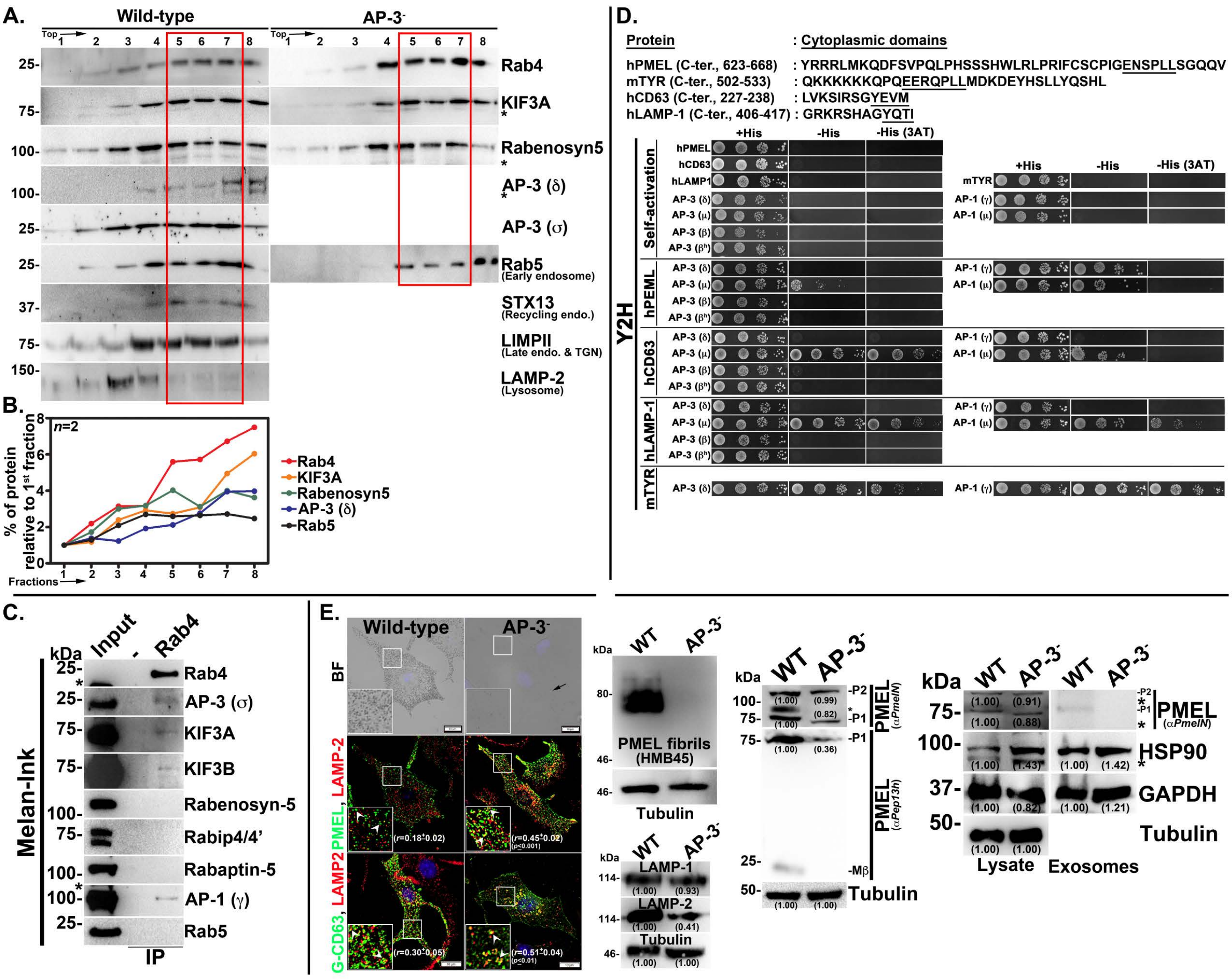

Fig. S4. Subcellular localization of Rab4A-AP-3-Rabenosyn-5- KIF3A complex in wild-type and AP-3⁻ melanocytes. Immunoprecipitation of Rab4A-complex in wild-type melanocytes. Y2H interaction between cargo tails and AP-3 or AP-1 subunits and analysis of PMEL trafficking in AP-3-deficient melanocytes. (A, B) Subcellular fractionation of wild-type and AP-3⁻ melanocytes and probed the fractions for localization of Rab4A-AP-3- Rabenosyn-5-KIF3A complex with respect to other organelle makers as indicated. Red box indicates the distribution of the molecules in different fractions. *, non-specific bands. Graph in B represents the percentage enrichment (relative to the 1st fraction) of Rab4A-AP-3-Rabenosyn-5-KIF3A complex in each fraction analyzed in wild-type melanocytes. (C) Immunoprecipitation of endogenous Rab4 in wild-type melanocytes. Both cell lysate (input) and IP blots were probed as indicated. *, non-specific bands. (D) List of C-terminal tails of cargo proteins used for studying the interaction with adaptor proteins. Conserved adaptor binding motifs in the protein sequence were underlined. The Y2HGold yeast strain was transformed with respective bait (cargo tails) and prey (AP-3 or AP-1 subunits) plasmids as shown in the figure and the transformants were selected and spotted on Y2H reporter activity plates. Self-activation of the plasmids was shown separately and the empty vectors were used as negative control (not shown) in the assay. (E) BF and IFM analysis of wild-type and AP-3-deficient melanocytes. Arrow indicates hypopigmentation of AP-3-deficient (AP-3⁻) melanocytes. Arrowheads point to the cargo localization to lysosomes. Nuclei are stained with Hoechst33258. The insets are a magnified view of the white boxed areas. The Pearson's coefficient (r) between the two markers was indicated separately (mean±s.e.m.). Scale bars, 10 µm. Immunoblotting analysis of PMEL fibrils and the proteins both in cell lysates and exosomes released by AP-3⁻ cells. Tubulin and HSP90 or GAPDH were used as a loading control for cell lysates and exosomes respectively. P1/P2 and Mβ, full length/glycosylated ER-form and processed PMEL bands. *, non-specific bands. Protein band intensities were quantified and indicated on the gels.

Supplemental movies: Respective cell types were transfected with GFP-Rab4A and RFP-STX13 constructs and imaged by live cell imaging for 5 min in a Zeiss LSM880 laser scanning microscope with Airyscan mode as described in the materials and methods. The image series were analyzed by using Zen lite 2.0 or ImageJ software. Movies were converted into 'avi' format using ImageJ and displayed at 3 fps (frames per second).

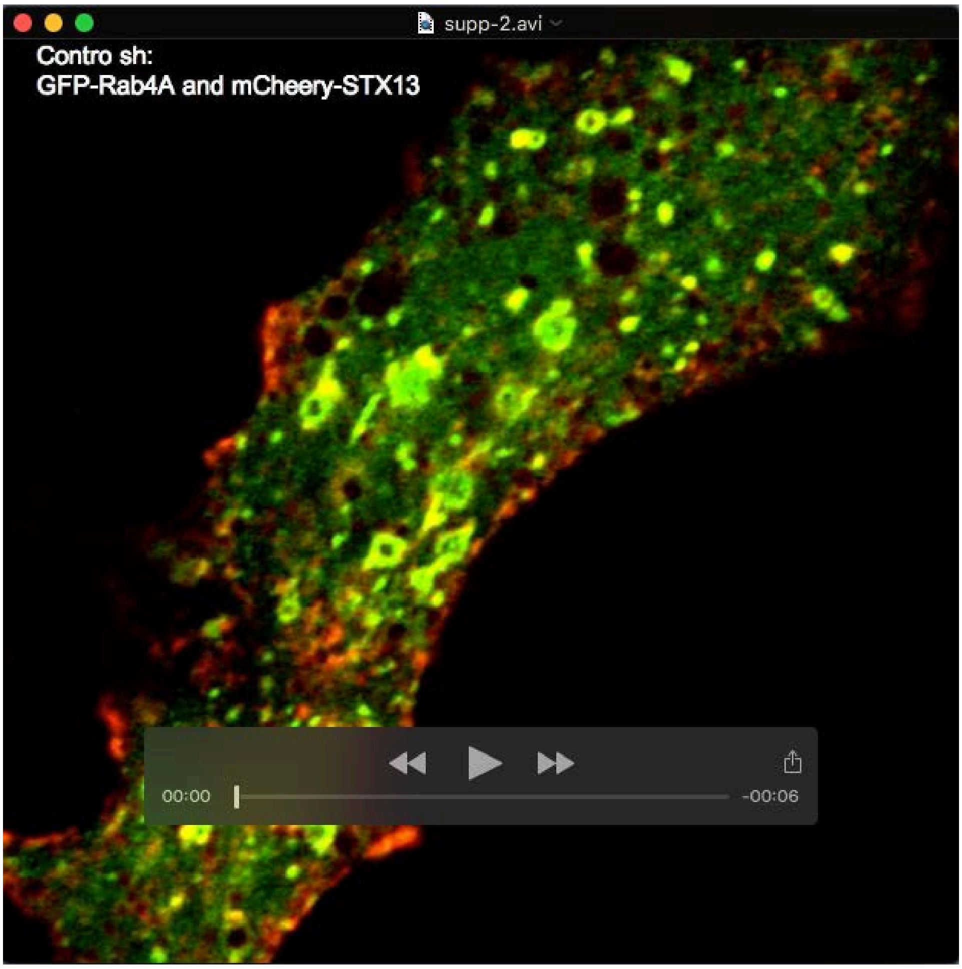

Movie S1. Time-lapse imaging of GFP-Rab4A and RFP-STX13 in wild-type (melan-Ink4a) melanocytes.

Journal of Cell Science • Supplementary information

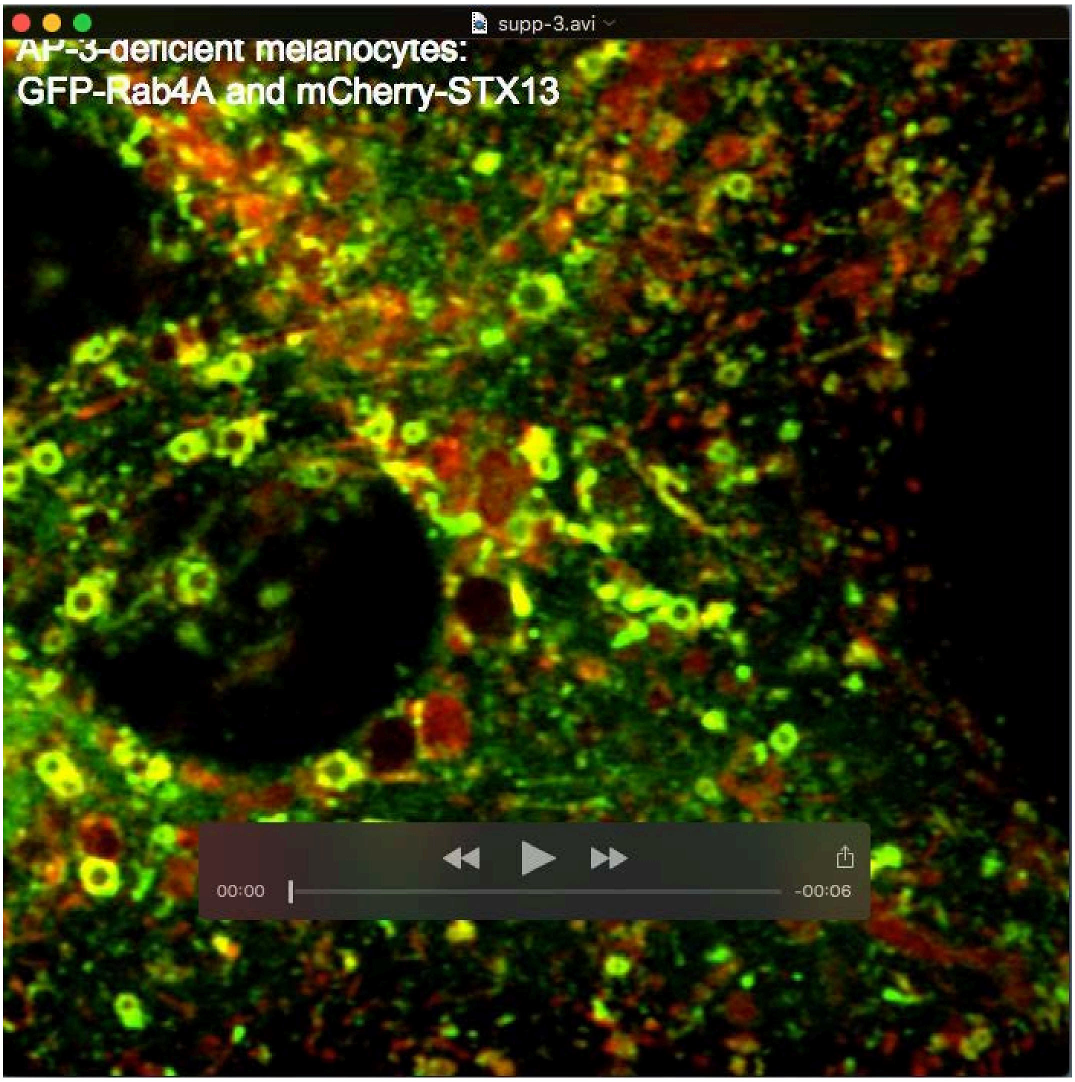

Movie S2. Time-lapse imaging of GFP-Rab4A and RFP-STX13 in AP-3-deficient (melan-mh) melanocytes.

Table S1. List of TRC shRNA plasmids (specific to human, h) and their target sequence

ShRNA	Target sequence in pLKO vector	% homology to mouse Rab
hRab3A sh	5'-GACCATCTATCGCAACGACAA-3'	95
hRab4A sh	5'-ACCTACAATGCGCTTACTAAT-3'	100
hRab4B sh	5'-CGCACTATCCTCAACAAGATT-3'	100
hRab5A sh	5'-GGCAAGCAAGTCCTAACATTG-3'	100
hRab5B sh	5'-GCAGATGACAACAGCTTATTG-3'	95
hRab5C sh	5'-CATCACCAACACAGATACATT-3'	100
hRab7A sh	5'-ATGGATAAATTGCCGTTATTT-3'	85
hRab11A sh	5'-ATCATGCTGATAGTAACATTG-3'	100

Table S2. List of retroviral shRNAs (specific to mouse, m) and their target sequence

ShRNA	Target sequence
mRab4A sh-1	5'-AAATGTCGGTGGTAAATATGT-3'
mRab4A sh-2	5'-AAGAGAATGAGCTGATGTTCC-3'
mRabenosyn-5 sh-1	5'- GACCCAAGGATATGAATCATT-3'
mRabenosyn-5 sh-2	5'- CCTCCCAGTTAAAGGAAGTAA-3'
mRabip4 sh-1	5'-GGTCTCTATGGAGTCATCTCCTCTA-3'
mRabip4 sh-2	5'- CCTCAAACATGGGCTGAAA-3'
mRabaptin-5 sh-1	5'- TTGCCACAGTCTCTGAGAATA-3'
mRabaptin-5 sh-2	5'-TCAAGCGGAACAGTGTTTAAA-3'
mKIF3A sh-1	5'-CCAAAGACATTTACTTTCGAT-3'
mKIF3B sh-2	5'-CCATTGGAAATTACATCCTAT-3'

Table S3. List of mouse specific primers used for transcript analysis

Gene	Forward Primer	Reverse primer	Size (bp)
GAPDH	5'-GAGCCAAACGGGTCATCATCT-3'	5'-GAGGGGCCATCCACAGTCTT-3'	220
RAB3A	5'-ATGGCTTCCGCCACAGAC-3'	5'-TCAGCAGGCACAATCCTG-3'	663
RAB4A	5'-ATGGCGCAGACCGCCATGTCC-3'	5'-CTAGCAGCCACACTCCTGTGC-3'	657
RAB4B	5'-ATGGCCGAGACCTACGACTTC-3'	5'-TCAGCAGCCACAGGGCTGAGG-3'	642
RAB5A	5'-ATGGCTAATCGAGGAGC-3'	5'-TCAGTTACTACAACACTG-3'	648
RAB5B	5'-ATGACTAGCAGAAGTACA-3'	5'-TCAGTTGCTACAACACTG-3'	648
RAB5C	5'-ATGGCGGGTCGAGGAGGT-3'	5'-TCAGTTGCTGCAGCACTG-3'	705
RAB7A	5'-ATGACCTCTAGGAAGAAA-3'	5'-TCAACAACTGCAGCTTTC-3'	624
RAB11A	5'-ATGGGCACCCGCGACGAC-3'	5'-TTAGATGTTCTGACAGCAC-3'	651

Table S4. List of yeast two-hybrid (Y2H) constructs used for protein-protein interaction

Bait plasmids	Prey plasmids
pGBKT7 (empty vector control)<br>pGBKT7 – hPMEL$^{623\text{-}668}$<br>pGBKT7 – hCD63$^{227\text{-}238}$<br>pGBKT7 – hLAMP-1$^{406\text{-}417}$<br>pGBKT7 - mTYR$^{502\text{-}533}$	pGADT7 (empty vector control)<br>pGADT7 – AP-3(δ)<br>pACT2 – AP-3(μ3)<br>pGADT7 – AP-3(β3A)<br>pGADT7 – AP-3(β3A-hinge)<br>pGADT7 – AP-1(γ)<br>pACT2 – AP-1(μ1)
